# Design and synthesis of some new benzoylthioureido benzenesulfonamide derivatives and their analogues as carbonic anhydrase inhibitors

**DOI:** 10.1080/14756366.2022.2132485

**Published:** 2022-10-28

**Authors:** Khulood H. Oudah, Walaa R. Mahmoud, Fadi M. Awadallah, Azza T. Taher, Safinaz E.-S Abbas, Heba Abdelrasheed Allam, Daniela Vullo, Claudiu T. Supuran

**Affiliations:** a Pharmaceutical Chemistry Department, College of Pharmacy, Al-Ayen University, Nasiriyah, Iraq; b Pharmaceutical Chemistry Department, Faculty of Pharmacy, Cairo University, Cairo, Egypt; c Department of Pharmaceutical Organic Chemistry, Faculty of Pharmacy, Cairo University, Cairo, Egypt; d Department of Pharmaceutical Organic Chemistry, Faculty of Pharmacy, October 6 University(O6U), Giza, Egypt; e Department NEUROFARBA – Pharmaceutical and Nutraceutical section, University of Firenze, Università degli Studi di Firenze, Sesto Fiorentino, Italy

**Keywords:** Carbonic anhydrase, benzenesulfonamides, SLC-0111, zinc binding-group (ZBG)

## Abstract

The present investigation reports the design and synthesis of three series of benzoylthioureido derivatives bearing either benzenesulfonamide **7a–f**, benzoic acid **8a–f** or ethylbenzoate **9a–f** moieties. The synthesised compounds were screened for their carbonic anhydrase inhibitory activity (CAI) against four isoforms hCA I, II, IX, and XII. Compounds **7a, 7b, 7c**, and **7f** exhibited a potent inhibitory activity towards hCAI (*K*_i_s = 58.20, 56.30, 33.00, and 43.00 nM), respectively compared to acetazolamide **(AAZ)** and SLC-0111 (*K*_i_s = 250.00 and 5080.00 nM). Compounds **7a, 7b, 7c, 7e**, and **7f** elicited selectivity over h CA II (*K*_i_s = 2.50, 2.10, 56.60,39.60 and 39.00 nM) respectively, relative to **AAZ** and SLC-0111(*K*_i_s = 12.10 and 960.00 nM). Also, compounds **7c**, **7f,** and **9e** displayed selectivity against the tumour-associated isoform hCA IX (*K*_i_s = 31.20, 30.00 and 29.00 nM) respectively, compared to **AAZ** and SLC-0111 (*K*_i_s = 25.70 and 45.00 nM). Additionally, compounds **8a** and **8f** revealed a moderate to superior selectivity towards hCAXII (*K*_i_s = 17.00 and 11.00 nM) relative to **AAZ** and SLC-0111(*K*_i_s = 5.70 and 45.00 nM). Molecular docking and ADME prediction studies were performed on the most active compounds to shed light on their interaction with the hot spots of the active site of CA isoforms, in addition to prediction of their pharmacokinetic and physicochemical properties.

## Graphical Abstract

**Figure:**
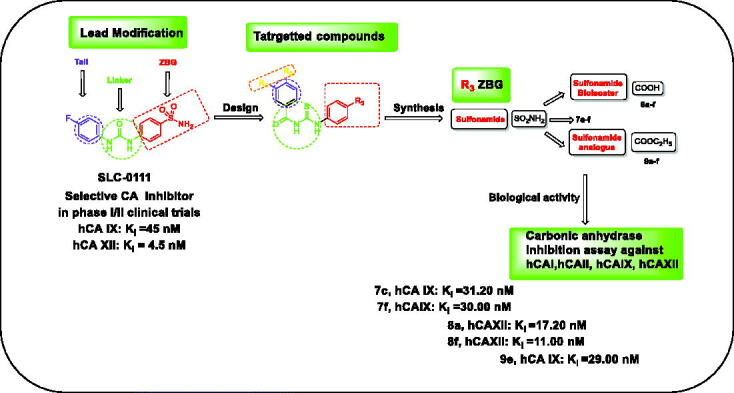

## Introduction

In the 1930s, the enzyme carbonic anhydrase (CAs, EC 4.2.1.1) was identified which are zinc metalloenzymes that catalyse the reversible hydration of CO_2_ and H_2_O to HCO_3_^−^ and H^+^ ions in all organisms (CO_2_ + H_2_O ⇌ HCO_3_^−^ +H^+^).^1^ Only the α-form is present in humans, however there are 15 isoforms of CA that have been identified, each with its own subcellular location. hCA I, III, VII, and XIII are cytosolic, hCA IV, IX, XII, and XIV are membrane bound; hCA VA and VB are mitochondrial; isoform VI is secreted; while CA VIII, X, and XI are catalytic. Cells can readily adjust the extracellular and intracellular pH and CO_2_/HCO_3_ pools by using any of these CA isoforms.^2^ Several metabolic reactions (such as lipogenesis, ureagenesis, and gluconeogenesis), pH and CO_2_ homeostasis, electrolyte secretion, respiration, bone resorption, and tumorigenicity, are all dependent on this CA-catalysed reaction^3^^,^^4^. Increased expression of CA isoforms in humans has been linked to a variety of illness conditions. Oedema of the retina and brain, glaucoma, epilepsy, and altitude sickness have all been associated to the cytosolic hCA I and II isoforms^5^. Some human isoenzymes, especially hCA IX and hCA XII, have been linked to cancer development. However, hCA IX and hCA XII are involved in the regulation of extracellular and intracellular pH, as well as the metabolism of tumour cells, so they are promising novel targets in anticancer drug research and development for the management of hypoxic tumours due to their overexpression in a variety of human malignancies^6^. Due to recent epidemic outbreaks, scientists have been looking for potent compounds that can affect a wide range of diseases and designing them to be used in a variety of targets. Sulphonamide is one of these classes, with applications ranging from antibiotics to current anti-cancer therapy^7^^,^^8^. The sulfamoyl group, which binds to the Zn^+2^ ion at the active site, certainly, many heterocyclic based sulphonamides are the most versatile scaffolds used to make selective and potent CA inhibitors, and they play a key role in CA inhibition^9–12^.

**Figure 1.**
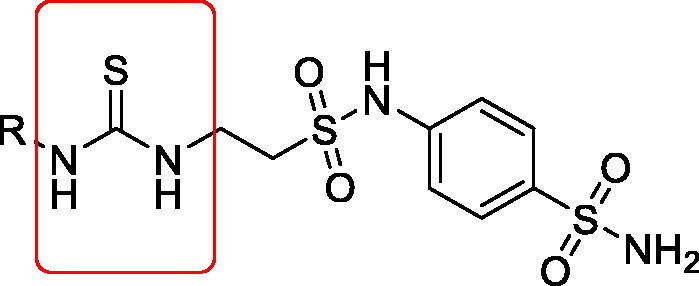
General structure of taurine sulphonamides terminated with thioureido tails^15^.

Because of its ability to form many stable hydrogen bonds with targeted protein, the ureido moiety is one of the most prevalent functional groups within medications of synthetic or natural origin^13^^,^^14^. For N,N-alkylated compounds of the class reported by Zhang^15^ and Liguori et al.,^16^ the significant function of the ureido moiety in promoting the selectivity of carbonic anhydrase inhibitors (CAIs) was clearly demonstrated. On the other hand, Akgul et al.^17^^,^^18^. demonstrated that thioureido moiety, a bioisoster for ureido, is another potent scaffold for CAIs (Figure 1).

In recent years, many approaches have been used to synthesise effective and selective carbonic anhydrase inhibitors. “Tail” approach is the most popular one, in which a flexible linker is used to attach tails of various chemical nature to an aromatic/heterocyclic ring with a zinc binding group (ZBG), such as primary sulfamoyl and carboxylic acid functionalities^19^. Another approach is the addition of multiple ''tails’' to the aromatic sulphonamide moiety to improve interaction with hydrophobic or hydrophilic portions of the active site consequently it can interact with non-traditional amino acid residues on the middle and outer regions of the active site cavity, resulting in better ligand binding and isozyme selectivity, this is called the ‘two-tail’ approach^20^.

**Figure 2.**
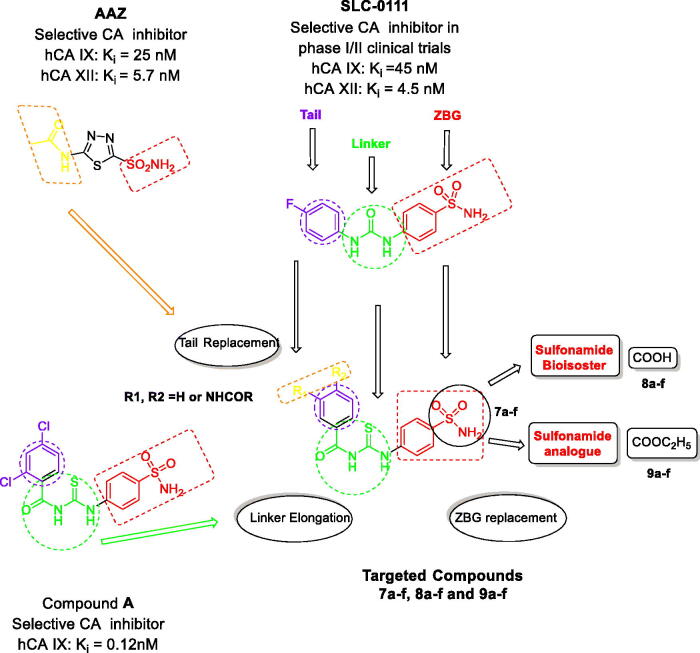
The design for the targeted compounds benzoylthioureido benzenesulfonamide derivatives (**7a–f**) and their analogues (**8a–f**) and (**9a–f**).

SLC-0111, is the first ureidobenzene sulphonamide CAI, currently in phase I/II clinical studies for the treatment of advanced hypoxic solid tumours with CA IX and XII overexpression. It was discovered by using ‘tail’ approach. SLC-0111 has high selectivity towards IX, XII isoforms^6^. Additionally, the benzoylthioureido benzene sulphonamide derivative compound **A**^18^ (Figure 2) is reported as a highly potent and selective inhibitor of CA IX (IC_50_ = 0.12 nM). Based on the aforementioned facts, this work suggests the synthesis of some novel benzoylthioureido benzenesulfonamides and their analogues as promising carbonic anhydrase inhibitors.

The present study includes several approaches to design the targeted compounds. This is achieved by replacement of the substituted phenyl moiety of SLC-0111 and compound **A** with substituted benzamide ones either by keeping the acetamido moiety of AAZ or incorporating other substituted amide, maintaining the carbonyl thioureido linker of compound **A** or spacer elongation of the ureido linker in SLC-0111 along with retaining the benzenesulfonamide moiety to afford compounds **7a–f**.

Based on the bioisosteric replacement strategy promoted by numerous research organizations^18^^,^^21–24^, the zinc binding group (sulfamoyl) of **7a–f** is replaced by its carboxylic acid bioisostere to give **8a–f**.

Furthermore, structural modification involves replacement of the sulfamoyl moiety with ethylcarboxylate as prodrug to the respective carboxylic acid group to obtain **9a–f**.

All the newly synthesised compounds **7a–f**, **8a–f**, and **9a–f** were characterised and biologically evaluated towards a panel of hCA I, II, IX, and XII isoforms.

## Materials and methods

### Chemistry

Starting materials, solvents, and reagents were purchased from Sigma-Aldrich and used without further purification. Melting points were recorded on a Stuart SMP10 digital melting point apparatus and were uncorrected. The purities of compounds were monitored by analytical TLC, performed on silica gel 60 F254 packed on Aluminium sheets, purchased from Merck, with visualisation under UV light (254 nm). Infra-red (IR) Spectra were recorded as KBr discs using a Shimadzu FT-IR 8400S infra-red spectrophotometer. ^1^H NMR and ^13^C NMR spectra were determined in DMSO-*d_6_* and recorded on 400 MHz spectrophotometer for ^1^H NMR and 100 MHz spectrophotometer for ^13^C NMR (Bruker AG, Switzerland) at Faculty of Pharmacy, Cairo University; chemical shift (*δ*) values are expressed in parts per million (ppm) and coupling constants (*J*) in Hertz (Hz). The abbreviations used are as follows: s, singlet; d, doublet; m, multiplet. Elemental microanalyses and mass spectra were performed at the Regional Centre for Mycology and Biotechnology, Al-Azhar University, Egypt.

Compounds **3a–f**, **4a–f**, and **5a–f** have been synthesised as reported^25–38^.

#### General procedure for the preparation of compounds 7a–f

A solution of freshly prepared benzoyl chloride derivatives **4a–f** (1 mmol) and ammonium thiocyanate (0.08 g/1 mmol) in acetone (10 ml) was heated under reflux for 1–3 h. After completion of reaction (monitored by TLC), the reaction mixture was cooled to room temperature and the formed precipitate (NH_4_Cl) was filtered off. To the freshly prepared solution of appropriate benzoyl isothiocyanate derivative **5a–f**, sulphanilamide **6a** (0.17 g/1 mmol) was added and the mixture was stirred under reflux for 2–3 h. Then the reaction was cooled to room temperature, the formed precipitate was collected by filtration and recrystallized from ethanol to give the pure product **7a–f**.

##### 3-Acetamido-N-((4-sulfamoylphenyl)carbamothioyl)benzamide 7a

Brown crystals, (yield 80%), m.p. 256–258 °C; IR (KBr, ν_max_/cm^−1^): 3430–3342 (NHs), 1660, 1610 (2C = O), 1350 & 1190 (SO_2_); ^1^H NMR δ 2.09 (s, 3H, CH_3_), 7.40 (s, 2H, D_2_O exchangeable, -SO_2_NH_2_), 7.45 (t, *J* = 7.9 Hz, 1H, Ar-H), 7.64 (d, *J* = 7.6 Hz, 1H, Ar-H), 7.84 − 7.87 (m, 3H, Ar-H), 7.91 (d, *J* = 8.7 Hz, 2H, Ar-H), 8.18 (s, 1H, Ar-H), 10.20 (s, 1H, D_2_O exchangeable, -NH),11.66 (s, 1H, D_2_O exchangeable, -NH),12.66 (s, 1H, D_2_O exchangeable, -NH); Anal. Calcd. for C_16_H_16_N_4_O_4_S_2_ (392.45): C, 48.97; H, 4.11; N, 14.28; Found C, 49.15; H, 4.29; N, 14.35.

##### 3-Isobutyramido-N-((4-sulfamoylphenyl)carbamothioyl)benzamide 7b

Light brown crystals, (yield 76%), m.p. 240–242 °C; IR (KBr, ν_max_/cm^−1^): 3435–3329 (NHs), 1659, 1615 (2C = O), 1340 & 1170 (SO_2_); ^1^H NMR δ 1.12 (d, *J* = 6.8 Hz, 6H, CH(CH_3_)_2_), 2.60–2.66 (m, 1H, CH(CH_3_)_2_), 7.40 (s, 2H, D_2_O exchangeable, -SO_2_NH_2_), 7.45 (t, *J* = 7.9 Hz, 1H, Ar-H), 7.63 (d, *J* = 7.9 Hz, 1H, Ar-H), 7.85–7.93 (m, 5H, Ar-H), 8.21 (s, 1H, Ar-H), 10.09 (s, 1H, D_2_O exchangeable, -NH),11.67 (s, 1H, D_2_O exchangeable, -NH), 12.66 (s, 1H, D_2_O exchangeable, -NH). ^13^C NMR *δ*: 19.92 (CH(CH_3_)_2_), 35.46 (CH(CH_3_)_2_), 119.74, 123.53, 124.00, 124.89, 126.73, 129.30, 133.16, 140.01, 141.38, 141.81, 168.65 (C = O), 176.02 (C = O), 179.85 (C = S); MS (*m/z*): 420.83 [M]^+^; Anal. Calcd. for C_18_H_20_N_4_O_4_S_2_ (420.50): C, 51.41; H, 4.79; N, 13.32; Found C, 51.70; H, 4.88; N, 13.57

##### 3-(4-Methylbenzamido)-N-((4-sulfamoylphenyl)carbamothioyl)benzamide 7c

White crystals, (yield 80%), m.p. 210–212 °C; IR (KBr, ν_max_/cm^−1^): 3425–3336 (NHs), 1681, 1651 (2C = O), 1330 & 1157 (SO_2_); ^1^H NMR *δ* 2.41 (s, 3H, CH_3_), 7.36 (d, *J* = 7.9 Hz, 2H, Ar-H), 7.41 (s, 2H, D_2_O exchangeable, -SO_2_NH_2__,_), 7.51 (t, *J* = 8.0 Hz, 1H, Ar-H), 7.71 (d, *J* = 7.8 Hz, 1H, Ar-H), 7.85 (d, *J* = 8.8 Hz, 2H, Ar-H), 7.91–7.94 (m, 4H, Ar-H), 8.06 (d, *J* = 7.9 Hz, 1H, Ar-H), 8.42 (s, 1H, Ar-H), 10.42 (s, 1H, s, 1H, D_2_O exchangeable, -NH), 11.68 (s, 1H, s, 1H, D_2_O exchangeable, -NH), 12.68 (s, 1H, D_2_O exchangeable, -NH). ^13^C NMR *δ* 21.25 (CH_3_), 120.71, 123.87, 124.66, 124.97, 126.49, 127.96, 129.01, 129.22, 131.90, 132.84, 139.63, 141.15, 141.56, 142.13, 165.81 (C = O), 168.36 (C = O), 179.60 (C = S); MS (*m/z*): 468.24 [M]^+^; Anal. Calcd. for C_22_H_20_N_4_O_4_S_2_ (468.55): C, 56.40; H, 4.30; N, 11.96; Found C, 56.63; H, 4.28; N, 12.19.

##### 4-Acetamido-N-((4-sulfamoylphenyl)carbamothioyl)benzamide 7d

*Light yellow crystals, (yield 75%),* m.p. 218–220 °C; IR (KBr, ν_max_/cm^−1^): 3380–3275 (NH_s_), 1675, 1614 (C = O), 1540 (C = S), 1345 &1185 (SO_2_); ^1^H NMR *δ* 2.09 (s, 3H, CH_3_), 7.40 (s, 2H, D_2_O exchangeable, -SO_2_NH_2_), 7.44 (d, *J* = 8.0 Hz, 1H, Ar-H), 7.64 (d, *J* = 7.6 Hz, 1H, Ar-H), 7.83 − 7.93 (m, 6H, Ar-H), 10.19 (s, 1H, D_2_O exchangeable, -NH),11.66 (s, 1H, D_2_O exchangeable, -NH), 12.65 (s, 1H, D_2_O exchangeable, -NH); Anal. Calcd. for C_16_H_16_N4O_4_S_2_ (392.45): C, 48.97; H, 4.11; N, 14.28; Found C, 49.13; H, 4.07; N, 14.15.

##### 4-Isobutyramido-N-((4-sulfamoylphenyl)carbamothioyl)benzamide 7e

*White crystals, (yield 79%),* m.p. 248–250 °C; IR (KBr, ν_max_/cm^−1^): 3375–3271 (NHs), 1670, 1650 (C = O) 1338 & 1161 (SO_2_); ^1^H NMR *δ* 1.12 (d, *J* = 6.7 Hz, 6H,-CH(CH_3_)_2_), 2.63–2.66 (m, 1H, -CH(CH_3_)_2_), 7.40 (s, 2H, D_2_O exchangeable, -SO_2_NH_2_), 7.76 (d, *J* = 8.5 Hz, 2H, Ar-H), 7.84 (d, *J* = 8.4 Hz, 2H, Ar-H), 7.91 (d, *J* = 8.4 Hz, 2H, Ar-H), 7.99 (d, *J* = 8.5 Hz, 2H, Ar-H), 10.22 (s, 1H, D_2_O exchangeable, -NH), 11.52 (s, 1H, D_2_O exchangeable, -NH), 12.81 (s, 1H, D_2_O exchangeable, -NH); ^13^C NMR *δ* 19.85 (-CH(CH_3_)_2_), 35.57 (-CH(CH_3_)_2_), 118.67, 124.79, 126.08, 126.74, 130.49, 141.40, 141.74, 144.49, 167.87 (C = O), 176.40 (C = O), 179.96 (C = S); MS (m/z): 420.03 [M]^+^; Anal. Calcd. for C_18_H_20_N_4_O_4_S_2_ (420.50): C, 51.41; H, 4.79; N, 13.32; Found C, 51.69; H, 4.85; N, 13.54.

##### 4-Methyl-N-(4(((4sulfamoylphenyl)carbamothioyl)carbamoyl)phenyl) benzamide 7f

*Yellow crystals, (yield 82%),* m.p. 228–230 °C; IR (KBr, ν_max_/cm^−1^): 3350–3268 (NHs), 1680, 1650 (2C = O), 1320 & 1116 (SO_2_); ^1^H NMR *δ* 2.41 (s, 3H,CH_3_), 7.37 (d, *J* = 7.9 Hz, 3H, Ar-H), 7.42 (s, 2H, D_2_O exchangeable, -SO_2_NH_2_), 7.78–7.86 (m, 3H,Ar-H), 7.88–7.95 (m, 6H, Ar-H), 10.49 (s, 1H, D_2_O exchangeable, -NH), 11.63 (s, 1H, D_2_O exchangeable, -NH), 12.78 (s, 1H, D_2_O exchangeable, -NH).; ^13^C NMR *δ;* 21.34 (CH_3_), 1224.58, 126.51, 126.74, 129.08, 129.23, 129.30, 129.35, 131.25, 141.16, 141.54, 144.01, 165.96 (C = O), 168.24 (C = O), 179.71 (C = S); Anal. Calcd. for C_22_H_20_N_4_O_4_S_2_ (468.55): C, 56.40; H, 4.30; N, 11.96; Found C, 56.57; H, 4.24; N, 12.23.

#### General procedure for the preparation of compounds 8a–f

A solution of freshly prepared benzoyl chloride derivatives **4a–f** (1 mmol) and ammonium thiocyanate (0.08 g/1 mmol) in acetone (10 ml) was heated under reflux for 1–3 h. After completion of reaction (monitored by TLC), the reaction mixture was cooled to room temperature and the formed precipitate (NH_4_Cl) was filtered off. To the freshly prepared solution of benzoyl isothiocyanate derivatives **5a–f**, 4-aminobenzoic acid **6b** (0.014 g/1 mmol) was added and the mixture was stirred under reflux for 2–3 h. The reaction mixture was cooled and the resulting precipitate was collected by filtration and recrystallized from ethanol to give the pure products **8a–f**.

##### 4-(3-(3-Acetamidobenzoyl)thioureido)benzoic acid 8a

Brown crystals, (yield 76%), m.p. 247–249 °C; IR (KBr, ν_max_/cm^−1^): 3286 (br, NHs), 2672–2558 (OH carboxylic), 1720, 1680, 1630 (3C = O); ^1^H NMR *δ* 2.09 (s, 3H, CH_3_), 7.44 (t, *J* = 7.9 Hz, 1H, Ar-H), 7.64 (d, *J* = 7.92 Hz, 1H, Ar-H), 7.84 (d, *J* = 8.1 Hz, 1H, Ar-H), 7.89 (d, *J* = 8.6 Hz, 2H, Ar-H), 7.98 (d, *J* = 8.6 Hz, 2H, Ar-H), 8.17 (s, 1H, Ar-H), 10.20 (s, 1H, D_2_O exchangeable, -NH), 11.63 (s, 1H, D_2_O exchangeable, -NH), 12.72 (s, 1H, D_2_O exchangeable, -NH), 12.93 (s, 1H, D_2_O exchangeable, -OH). ^13^C NMR *δ* 24.47 (CH_3_), 119.57, 123.54, 123.84, 124.01, 128.55, 129.32, 130.39, 133.18, 139.91, 142.41, 167.17 (C = O), 168.62 (C = O), 169.13 (C = O), 179.43 (C = S); MS (*m/z*): 357.25 [M]^+^; Anal. Calcd. for C_17_H_15_N_3_O_4_S (357.38): C, 57.13; H, 4.23; N, 11.76; Found C, 56.82; H, 4.18; N, 11.69.

##### 4-(3-(3-Isobutyramidobenzoyl)thioureido)benzoic acid 8b

Light brown crystals, (yield 76%), m.p. 240–242 °C; IR (KBr, ν_max_/cm^−1^): 3286 (br, NHs), 2661–2548 (OH carboxylic), 1710, 1681, 1589 (3C = O); ^1^H NMR *δ* 1.12 (d, *J* = 6.4 Hz, 6H, CH(CH3)2), 2.59–2.64 (m, 1H, CH(CH_3_)_2_), 7.30 (d, *J* = 8.5 Hz, 1H, Ar-H), 7.44 (t, *J* = 8.0 Hz, 1H, Ar-H), 7.63 (d, *J* = 8.2 Hz, 1H, Ar-H), 7.89 − 8.00 (m, 4H, Ar-H), 8.20 (s, 1H, Ar-H) 10.08 (s, 1H, D2O exchangeable, -NH),11.63 (s, 1H, D2O exchangeable, -NH), 12.72 (s, 1H, D_2_O exchangeable, -NH), 12.97 (s, 1H, D_2_O exchangeable,-OH). ^13^C NMR *δ* 19.89 (CH(CH_3_)2), 31.03 (CH(CH_3_)2), 119.76, 124.04, 129.98, 130.41, 131.13, 131.44, 140.00, 142.41, 145.68, 167.19 (C = O), 176.05 (C = O), 176.50 (C = O), 179.46 (C = S); MS (*m/z*): 385.44 [M]^+^, 385.89 [M]^+^; Anal. Calcd. for C_19_H_19_N_3_O_4_S (385.44): C, 59.21; H, 4.97; N, 10.90; Found C, 59.44; H, 5.13; N, 11.08.

##### 4-(3-(3-(4-Methylbenzamido)benzoyl)thioureido)benzoic acid 8c

White crystals, (yield 78%), m.p. 254–256 °C; IR (KBr, ν_max_/cm^−1^): 3286 (br, NHs), 2665–2550 (OH carboxylic), 1710, 1655, 1615 (3C = O); ^1^H NMR *δ* 2.41 (s, 3H, CH_3_), 7.36 (d, *J* = 8.8 Hz, 2H, Ar-H), 7.51 (t, *J* = 7.9 Hz, 1H, Ar-H), 7.71 (d, *J* = 8.4 Hz, 1H, Ar-H), 7.88–8.07 (m, 7H, Ar-H), 8.41 (s, 1H, Ar-H), 10.42 (s, 1H, D_2_O exchangeable, -NH), 11.65 (s, 1H, D_2_O exchangeable, -NH), 12.74 (s, 1H, D_2_O exchangeable, -NH), 12.94 (s, 1H, D_2_O exchangeable,-OH); Anal. Calcd. for C_23_H_19_N_3_O_4_S (433.48): C, 63.73; H, 4.42; N, 9.69; Found C, 63.50; H, 4.63; N, 9.93.

##### 4-(3-(4-Acetamidobenzoyl)thioureido)benzoic acid 8d

Brown crystals, (yield 82%), m.p. 245–247 °C; IR (KBr, ν_max_/cm^−1^): 3185 (br, NHs), 2680–2530 (OH carboxylic), 1675, 1600 (3C = O); ^1^H NMR *δ* 2.10 (s, 3H, CH_3_), 7.73 (d, *J* = 8.5 Hz, 2H, Ar-H), 7.90 (d, *J* = 8.4 Hz, 1H, Ar-H), 7.92 − 7.94 (m, 4H, Ar-H), 7.95 (d, *J* = 8.5 Hz, 1H, Ar-H), 10.26 (s, 1H, D_2_O exchangeable, -NH),10.41 (s, 1H, D_2_O exchangeable, -NH),12.74 (s, 1H, D_2_O exchangeable, -NH),13.03 (s, 1H, D_2_O exchangeable,-OH). ^13^C NMR *δ* 21.82 (CH_3_), 111.58, 118.12, 131.20, 131.57, 131.75, 131.84, 132.34, 132.63, 162.65 (C = O), 164.32 (C = O), 168.53 (C = O), 177.67. (C = S); MS (*m/z*): 357.08 [M]^+^; Anal. Calcd. for C_17_H_15_N_3_O_4_S (357.38): C, 57.13; H, 4.23; N, 11.76; Found C, 57.18; H, 4.20; N, 11.73.

##### 4-(3-(4-Isobutyramidobenzoyl)thioureido)benzoic acid 8e

Yellow Crystals, (yield 76%), m.p. 240–242 °C; IR (KBr, ν_max_/cm^−1^):, 3190 (br NHs), 2665–2548 (OH carboxylic), 1685, 1604 (C = O); ^1^H NMR *δ* 1.14 (d, *J* = 6.8 Hz, 6H,CH(CH_3_)2), 2.63–2.69 (m, 1H, CH(CH_3_)2), 7.77 (d, *J* = 9.6, 2H, Ar-H), 7.90 − 7.98 (m, 6H, Ar-H), 10.22 (s, 1H, D_2_O exchangeable, -NH),11.50 (s, 1H, D_2_O exchangeable, -NH),12.88 (s, 1H, D_2_O exchangeable, -NH), 12.96 (s, 1H, D_2_O exchangeable,-OH). ^13^C NMR *δ* 19.84 (CH_3_), 35.57 (CH), 118.66, 123.93, 126.08, 128.49, 130.40, 130.47, 142.43, 144.47, 167.17 (C = O), 167.90 (C = O), 176.40 (C = O), 179.56 (C = S); MS (*m/z*): 385.90 [M]^+^; Anal. Calcd. for C_19_H_19_N_3_O_4_S (385.44): C, 59.21; H, 4.97; N, 10.90; Found C, 59.43; H, 5.08; N, 11.18.

##### 4-(3-(4–(4-Methylbenzamido)benzoyl)thioureido)benzoic acid 8f

White crystals, (yield 78%), m.p. 254–256 °C; IR (KBr, ν_max_/cm^−1^):, 3414, 3309 (NHs), 2669–2553 (OH carboxylic), 1683, 1681 (C = O); ^1^H NMR *δ* 2.41 (s, 3H, CH_3_), 7.36 (d, *J* = 7.9 Hz, 2H, Ar-H), 7.90 (d, *J* = 8.1 Hz, 4H, Ar-H), 7.96–8.00 (m, 4H, Ar-H), 8.04 (d, *J* = 8.8 Hz, 2H, Ar-H), 10.51 (s, 1H, D_2_O exchangeable, -NH), 11.54 (s, 1H, D_2_O exchangeable, -NH), 12.88 (s, 1H, D_2_O exchangeable, -NH), 12.96 (s, 1H, D_2_O exchangeable,-OH). ^13^C NMR *δ* 21.52 (CH_3_), 119.73, 123.94, 126.65, 128.34, 128.50, 129.48, 130.34, 130.40, 132.08, 142.44, 142.56, 144.37, 166.31 (C = O), 167.17 (C = O), 167.94 (C = O), 179.56 (C = S); MS (*m/z*): 433.31[M] ^+^; Anal. Calcd. for C_23_H_19_N_3_O_4_S (433.48): C, 63.73; H, 4.42; N, 9.69; Found C, 63.50; H, 4.63; N, 9.93.

#### General procedure for the preparation of compounds 9a–f

A solution of freshly prepared benzoyl chloride derivatives **4a–f** (1 mmol) and ammonium thiocyanate (0.08 g/1 mmol) in acetone (10 ml) was heated under reflux for 1–3 h. After completion of reaction (monitored by TLC), the reaction mixture was cooled to room temperature and the formed precipitate (NH_4_Cl) was filtered off. To the freshly prepared solution of benzoyl isothiocyanate derivative **5a–f**, ethyl 4-aminobenzoate **6c** (0.017 g/1 mmol) was added and the mixture was stirred under reflux for 2–3 h. The reaction mixture was cooled and the resulting precipitate was filtered and recrystallized from ethanol to give the pure product **9a–f**.

##### Ethyl 4–(3-(3-acetamidobenzoyl)thioureido)benzoate 9a

Brown crystals, (yield 65%), m.p. 242–244 °C; IR (KBr, ν_max_/cm^−1^): 3350–3322 (NHs), 1710 (C = O) ester, 1668, 1620 (C = O) amide; ^1^H NMR *δ* 1.32 (t, *J* = 7.1 Hz, 3H, CH_2_CH_3_), 2.09 (s, 3H, CH_3_), 4.31 (q, *J* = 7.1 Hz, 2H, CH_2_CH_3_), 7.45 (t, *J* = 7.9 Hz, 1H, Ar-H), 7.64 (d, *J* = 7.9 Hz, 1H, Ar-H), 7.84 (d, *J* = 8.1 Hz, 1H, Ar-H), 7.93 (d, *J* = 8.6 Hz, 2H, Ar-H), 8.00 (d, *J* = 8.7 Hz, 2H, Ar-H), 8.17 (s, 1H, Ar-H), 10.19 (s, 1H, D_2_O exchangeable, -NH),11.64 (s, 1H, D_2_O exchangeable, -NH), 12.73 (s, 1H, D_2_O exchangeable, -NH). ^13^C NMR *δ* 14.65 (CH_3_ CH_2_), 24.48 (CH_3_), 61.21 (CH_3_ CH_2_), 119.57, 123.54, 123.84, 124.07, 127.58, 129.31, 130.21, 133.17, 139.92, 142.73, 165.59 (C = O), 168.61 (C = O), 169.10 (C = O), 179.45 (C = S); Anal. Calcd. for C_19_H_19_N_3_O_4_S (385.44): C, 59.21; H, 4.97; N, 10.90; Found C, 59.47; H, 5.09; N, 11.13.

##### Ethyl 4–(3-(3-isobutyramidobenzoyl)thioureido)benzoate 9b

Brown crystals, (yield 73%), m.p. 245–247 C; IR (KBr, ν_max_/cm^−1^): 3290 (br, NHs), 1697 (C = O) ester, 1666, 1589 (C = O) amide; ^1^H NMR *δ* 1.12 (d, *J* = 6.8 Hz, 6H, -CH(CH_3_)_2_), 1.32 (t, *J* = 7.1 Hz, 3H, -CH_2_CH_3_), 2.59–2.66 (m, 1H, CH(CH_3_)_2_), 4.30 (q, *J* = 7.1 Hz, 2H, -CH_2_CH_3_), 7.44 (t, *J* = 8.0 Hz, 1H, Ar-H), 7.63 (d, *J* = 7.9 Hz, 1H, Ar-H), 7.88– 7.95 (m, 3H, Ar-H), 8.00 (d, *J* = 8.7 Hz, 2H, Ar-H), 8.21 (s, 1H, Ar-H), 10.08 (s, 1H, D_2_O exchangeable, -NH), 11.65 (s, 1H, D_2_O exchangeable, -NH), 12.74 (s, 1H, D_2_O exchangeable, -NH). ^13^C NMR *δ* 14.48 (CH_3_ CH_2_), 19.75 (CH(CH_3_)_2_), 35.28 (CH(CH_3_)_2_), 61.06 (CH_3_ CH_2_), 119.53, 123.38, 123.93,128.63, 129.13, 130.06, 132.98, 135.09, 139.83, 142.56, 165.42 (C = O), 168.50 (C = O), 173.30 (C = O), 175.84 (C = S); MS (*m/z*): 413.13 [M]^+^; Anal. Calcd. for C_21_H_23_N_3_O_4_S (413.49): C, 61.00; H, 5.61; N, 10.16; Found C, 61.17; H, 5.85; N, 10.38.

##### Ethyl 4–(3-(3–(4-methylbenzamido)benzoyl)thioureido)benzoate 9c

White crystals, (yield 80%), m.p. 236–238 °C; IR (KBr, ν_max_/cm^−1^): 3278 (br, NHs), 1716 (C = O) ester, 1662, 1593 (C = O) amide; ^1^H NMR *δ* 1.35 (t, *J* = 7.1 Hz, 3H, -CH_2_CH_3_), 2.41 (s, 3H, CH_3_), 4.34 (q, *J* = 2.2 Hz, 2H, -CH_2_CH_3_), 7.35–7.37 (m, 3H, Ar-H), 7.51 (d, *J* = 7.8 Hz, 1H, Ar-H), 7.71 (d, *J* = 7.4 Hz, 1H, Ar-H), 7.91–8.08 (m, 6H, Ar-H), 8.42 (s, 1H, Ar-H), 10.43 (s, 1H, s, 1H, D_2_O exchangeable, -NH), 11.68 (s, 1H, s, 1H, D_2_O exchangeable, -NH), 12.76 (s, 1H, s, 1H, D_2_O exchangeable, -NH). ^13^C NMR *δ* 14.42 (CH_3_CH_2_), 21.27 (CH_3_), 60.98 (CH_3_CH_2_), 120.73, 123.85, 124.97, 127.36, 127.98, 129.08, 129.22, 129.28, 129.99, 131.93, 132.84, 139.67, 142.11, 142.50, 165.36 (C = O), 165.79 (C = O), 168.38 (C = O), 179.23 (C = S); MS (*m/z*): 461.26 [M + 1] ^+^; Anal. Calcd. for C_25_H_23_N_3_O_4_S (461.54): C, 65.06; H, 5.02; N, 9.10; Found C, 64.97; H, 5.18; N, 9.37.

##### Ethyl 4–(3-(4-acetamidobenzoyl)thioureido)benzoate 9d

Brown crystals, (yield 68%), m.p. 237–239 °C; IR (KBr, ν_max_/cm^−1^): 3345–3320 (NHs), 1700 (C = O) ester, 1675, 1635 (C = O) amide; ^1^H NMR *δ* 1.33 (t, *J* = 7.1 Hz, 3H, CH_2_CH_3_), 2.12 (s, 3H,CH_3_), 4.38 (q, *J* = 7.1 Hz, 2H,CH_2_CH_3_), 7.71–7.76 (m, 2H, Ar-H), 7.92 (d, *J* = 8.8 Hz, 2H, Ar-H), 7.97–8.03 (m, 4H, Ar-H), 10.34 (s, 1H, D_2_O exchangeable, -NH),11.52 (s, 1H, D_2_O exchangeable, -NH),12.91 (s, 1H, D_2_O exchangeable, -NH); Anal. Calcd. for C_19_H_19_N_3_O_4_S (385.44): C, 59.21; H, 4.97; N, 10.90; Found C, 59.43; H, 5.14; N, 11.17.

##### Ethyl 4–(3-(4-isobutyramidobenzoyl)thioureido)benzoate 9e

White crystals, (yield 75%), m.p. 245–247 C; IR (KBr, ν_max_/cm^−1^): 3275 (br, NHs), 1712 (C = O) ester, 1662, 1608 (C = O) amide; ^1^H NMR *δ* 1.12 (d, *J* = 6.8 Hz, 6H,-CH(CH_3_)_2_), 1.31 (t, *J* = 7.1 Hz, 3H, -CH_2_CH_3_), 2.61–2.68 (m, 1H, CH(CH_3_)_2_), 4.30 (q, *J* = 7.1 Hz, 2H, -CH_2_CH_3_), 7.76 (d, *J* = 8.8 Hz, 2H, Ar-H), 7.92–7.95 (m, 2H, Ar-H), 8.00 (d, *J* = 7.6 Hz, 4H, Ar-H), 10.21 (s, 1H, D_2_O exchangeable, -NH), 11.50 (s, 1H, D_2_O exchangeable, -NH), 12.90 (s, 1H, D_2_O exchangeable, -NH). ^13^C NMR *δ* 14.64 (CH_3_ CH_2_), 19.84 (CH(CH_3_)_2_), 35.56 (CH(CH_3_)_2_), 61.20 (CH_3_ CH_2_), 118.61, 123.98, 126.03, 127.46, 130.22, 130.50, 142.73, 144.47, 163.84 (C = O), 165.58 (C = O), 171.64 (C = O),176.39 (C = S); MS (*m/z*): 413.45 [M]^+^; Anal. Calcd. for C_21_H_23_N_3_O_4_S (413.49): C, 61.00; H, 5.61; N, 10.16; Found C, 60.78; H, 5.84; N, 10.40.

##### Ethyl 4–(3-(4–(4-methylbenzamido)benzoyl)thioureido)benzoate 9f

Light yellow crystals, (yield 80%), m.p. 237–239 °C; IR (KBr, ν_max_/cm^−1^): 3340 (br, NHs), 1705 (C = O) ester, 1658, 1597 (C = O) amide; ^1^H NMR *δ* 1.45 (t, *J* = 6.3 Hz, 3H, -CH_2_CH_3_), 2.45 (s, 3H, CH_3_), 4.47 (m, 2H, -CH_2_CH_3_), 7.38 (d, *J* = 7.9 Hz, 2H, Ar-H), 7.55 (d, *J* = 7.9 Hz, 1H, Ar-H), 7.71 (d, *J* = 7.9 Hz, 1H, Ar-H), 8.01–8.08 (m, 8H, Ar-H), 10.58 (s, 1H, s, 1H, D_2_O exchangeable, -NH), 11.84 (s, 1H, s, 1H, D_2_O exchangeable, -NH), 12.92 (s, 1H, s, 1H, D_2_O exchangeable, -NH); MS (*m/z*): 462.21 [M + 1]^+^; Anal. Calcd. for C_25_H_23_N_3_O_4_S (461.54): C, 65.06; H, 5.02; N, 9.10; Found C, 64.90; H, 5.23; N, 9.34.

### Carbonic anhydrase inhibition assay

An applied photophysics stopped-flow instrument has been used for assaying the CA catalysed CO_2_ hydration activity.^28^ Phenol red (at a concentration of 0.2 mM) has been used as indicator, working at the absorbance maximum of 557 nm, with 20 Mm Hepes (pH 7.5) as buffer, and 20 mM Na_2_SO_4_ (for maintaining constant the ionic strength), following the initial rates of the CA-catalysed CO_2_ hydration reaction for a period of 10–100 s. The CO_2_ concentrations ranged from 1.7 to 17 mM for the determination of the kinetic parameters and inhibition constants. For each inhibitor, at least six traces of the initial 5–10% of the reaction have been used for determining the initial velocity. The uncatalyzed rates were determined in the same manner and subtracted from the total observed rates. Stock solutions of inhibitor (0.1 mM) were prepared in distilled-deionized water and dilutions up to 0.01 nM were done thereafter with the assay buffer. Inhibitor and enzyme solutions were preincubated together for 15 min at room temperature prior to assay, in order to allow for the formation of the E-I complex. The inhibition constants were obtained by non-linear least-squares methods using PRISM 3 and the Cheng–Prusoff equation, as reported earlier,^29–32^ and represent the mean from at least three different determinations. All CA isoforms were recombinant ones obtained in-house as reported earlier^33–35^.

### Molecular modeling study

#### Molecular docking

Molecular docking of the promising candidates **7a**, **7b**, **7c**, **7f**, **8a**, **8f**, and **9e** into the three-dimensional X-ray structure of human carbonic anhydrase (hCA) was conducted using MOE software package 2019.0102, to shed light on the possible interactions of the designed compounds. The crystal structures of human carbonic anhydrase isoforms I, II, IX, and XII in complex with their corresponding inhibitor were downloaded from the protein data bank (PDB: 3WXH, 3HS4, 3IAI, and 1JDO, respectively)^39^. All bound water molecules with exception of the ones that appear to participate in the ligand–receptor interaction were eliminated. The non-necessary molecules and cofactors were eliminated from the protein and the polar hydrogen was added. A light energy minimisation step with tethering the heavy atoms was performed on the protein using the MMFF94 forcefield. Docking of the lowest energetic conformer for each compound into the active site of human carbonic anhydrase isoforms I, II, IX, and XII using the default settings in MOE and the docking scores (S) were calculated.

#### Physicochemical, ADME, and pharmacokinetic properties prediction

The Swiss Institute of Bioinformatics’ (SIB) free Swiss ADME online tool was employed to evaluate physicochemical properties and determine ADME descriptors, pharmacokinetic features, medicinal chemistry compatibility, and drug-like characteristics of the most powerful newly synthesised **7a**, **7b**, **7c**, **7f**, **8a**, **8f**, and **9e** compounds. ChemDraw Professional 19.0 was used to draw the ligands, which were exported in SMILE format and then uploaded to an online server for evaluation.

## Results and discussion

### Chemistry

**Scheme 1.**
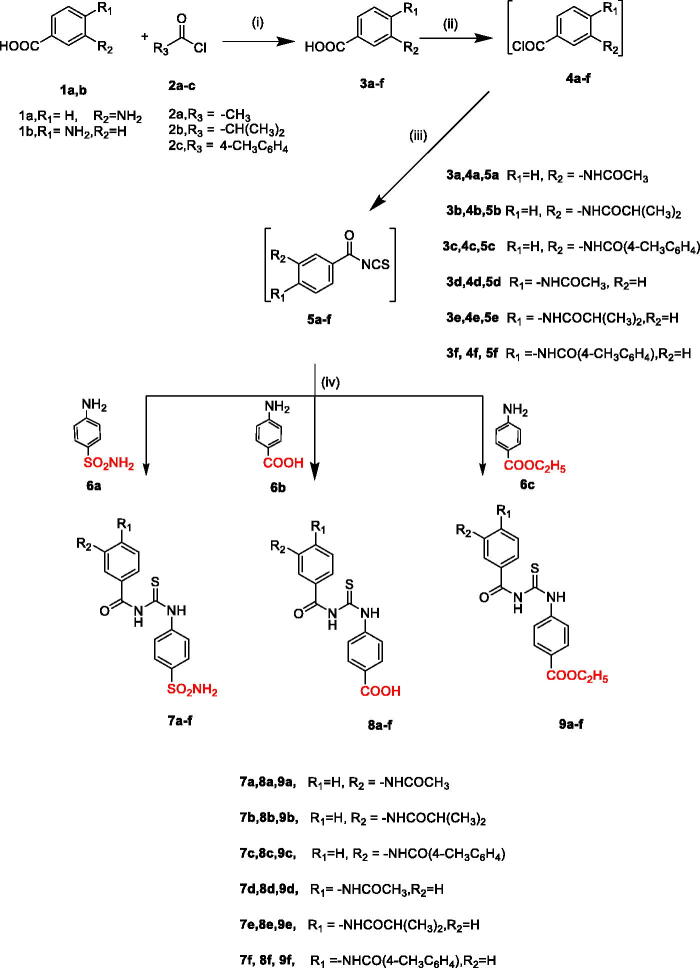
Synthetic pathways for compounds **7a–f**, **8a–f**, **9a–f**. Reagents and conditions: (i) KOH, acetonitrile, R.T, 1–2 h, (ii) SOCl_2_, methylene chloride, reflux, 4–5 h, (iii) NH_4_SCN, acetone, reflux, 1–3 h, (iv) acetone, reflux, 2–3 h.

Different substituted benzoylthioureido benzenesulfonamide derivatives and their analogues **7a–f**, **8a–f**, and **9a–f** were synthesised using the described synthetic methods as illustrated in Scheme 1. Acylation of the amino functionality of 3-amino or 4-amino benzoic acid **1a** or **1b** was performed using either acetyl chloride **2a**, isobutyryl chloride **2b** or 4-methylbenzoyl chloride **2c** in dry acetonitrile in the presence of potassium hydroxide to afford compounds **3a–f**, respectively. Then, *in situ* preparation of acid chloride derivatives **4a–f** was carried out by addition of thionyl chloride to the appropriate **3a–f** in methylene chloride under reflux. After removing thionyl chloride under vacuum, ammonium thiocyanate was added to the freshly prepared acid chlorides **4a–f** in dry acetone to give the key intermediate **5a–f**. Next, heating **5a–f** under reflux with sulphanilamide **6a** in dry acetone furnished **7a–f** in good yields (75–82%). IR spectra of compounds **7a–f** showed the appearance of characteristic bands at 3435–3268 cm^−1^ corresponding to NH_2_ and NH, in addition to two stretching vibration bands at 1320–1116 cm^−1^ attributed to the characteristic SO_2_ group.

^1^H NMR spectra revealed the appearance of D_2_O exchangeable signal in the aromatic region around 7.40 ppm corresponding to two protons of the SO_2_NH_2_ group as a singlet, along with D_2_O exchangeable three NH signals at about 10.08–12.81 ppm assigned to thioureido and benzamide protons. ^1^H NMR spectra of compounds **7a–f** elicited an increase in the integration of aromatic protons. Also, the aliphatic region of **7a** and **7d** showed the appearance of singlet signal at 2.09 ppm corresponding to (CH_3_) protons of acetamido moiety. The isopropyl derivatives **7b** and **7e** revealed the appearance of isopropyl protons as doublet at 1.12 ppm representing the two methyl protons and a multiplet at 2.60–2.66 ppm of the CH protons. Compounds **7c** and **7f**; showed a singlet signal at 2.41 ppm integrated for three protons of the tolyl moiety.

On the other hand, ^13^C NMR spectra of **7c** and **7f** displayed a signal at 21.25 and 21.34 ppm attributed to CH_3_ carbons of the tolyl moiety. The two carbonyl carbons and C = S carbon of **7a–f** appeared at 165–180 ppm.

Reacting the key intermediates **5a–f** with ethyl 4-aminobenzoic acid **6b** afforded **8a–f** in good yields 76–78%. IR spectra of compounds **8a–f** revealed the appearance of characteristic carboxylic OH stretching vibration bands in a wide range at nearly 2680–2530 cm^−1^. On the other hand, ^1^H NMR spectra showed the presence of an OH acidic exchangeable signal at 12.60–12.97 ppm, as well as three NH protons at 10.08–12.88 ppm. Also compounds **8a** and **8d** displayed the appearance of singlet signal at 2.09 and 2.10 ppm, respectively, corresponding to the (CH_3_) protons of the acetamido moiety. Compounds **8b** and **8e** were in accordance with their expected structures in ^1^HNMR by the appearance of isopropyl protons as doublet at 1.12 and 1.14 ppm, respectively, and multiplet signals at 2.59–2.69 ppm, whereas compounds **8c** and **8f**; elicited a singlet signal at 2.41 ppm integrated for the three protons of the tolyl (CH_3_) group. ^13^C NMR spectra of **8a** and **8d** showed signal at 24.47 and 21.82 ppm, respectively corresponding to (CH_3_) of acetamido group. Also, ^13^C NMR spectra of **8a-f** revealed the presence of additional carbonyl carbon of acid.

On the other hand, reacting **5a–f** with ethyl 4-aminobenzoate **6c** gave compounds **9a–f** in good yields 65–80%. IR spectra showed characteristic bands at 3350–3275 cm^−1^ corresponding to the NH groups along with carbonyl bands of the ester at range of 1697–1716 cm^−1^. ^1^H NMR spectra of **9a–f** elicited three exchangeable NH signals at 10.08–12.92 ppm, in addition to an increase in the aromatic region integration. Compounds **9a** and **9d**; revealed a singlet signal at 2.09, 2.12 ppm integrated for the three protons of the acetamido group. On the other hand, the isopropyl derivatives **9b** and **9e** showed the appearance of isopropyl protons as doublet at 1.12 ppm and multiplet signals at range 2.59–2.68 ppm. Moreover, compounds **9c** and **9f**; showed a singlet signal at 2.41, 2.45 ppm, respectively, integrated for the three protons of the tolyl (CH_3_) group. This is in addition to the usual triplet-quartet pattern of the ethyl group.

Further, ^13^C NMR spectra of **9a** displayed ethyl carbons (CH_3_CH_2_) at 14.65 ppm, (CH_3_CH_2_) carbon at 61.21 and CH_3_ acetamido carbon at 24.48 ppm along with two carbonyl carbons at 165.59 and 168.61 ppm and CS carbon at 179.45 ppm. ^13^C NMR spectra of **9b** and **9e** revealed the isopropyl carbons (CHCH_3_)2 at 19.75 and 19.84 ppm, respectively and (CHCH_3_)2 carbon at 35.28 and 35.56 ppm respectively, whereas, the tolyl (CH_3_) carbon of **9c** appeared at 21.27 ppm.

### Carbonic anhydrase inhibitory activity

All synthesised compounds **7a–f**, **8a–f**, and **9a–f** were tested using the standard inhibitor acetazolamide (AAZ) in a stopped flow CO_2_ hydrase assay for their capacity to suppress the physiologically relevant hCA isoforms, hCA I, II, IX, and XII^40^. The selection of these four isoforms was based on the fact that hCA II is antiglaucoma medication target^41^ while hCA IX and XII have been validated as targets for the treatment and prognosis of hypoxic malignancies^42^^,^^43^. Otherwise, hCA I is one of the most important off-target isoforms for antiglaucoma and anticancer CAI therapeutic applications^5^^,^^10^. The inhibitory data presented in Table 1 can be used to create the structural activity relationship (SAR) shown below.

**Table 1.** Carbonic anhydrase inhibitory activity of compounds **7a–f**, **8a–f**, and **9a–f** and the standard sulphonamide inhibitor acetazolamide (AAZ) using a stopped flow CO_2_ hydrase assay. 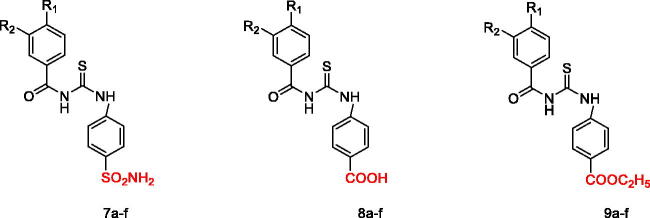

Compound No.	R_1_	R_2_	K_i_* (nM)			
			hCA I	hCA II	hCA IX	hCA XII
**7a**	H	-NHCOCH_3_	58.20	2.50	237.50	>100 000
**7b**	H	-NHCOCH(CH_3_)_2_	56.30	2.10	149.30	>100 000
**7c**	H	-NHCO(4-CH_3_C_6_H_4_)	33.00	56.60	31.20	>100 000
**7d**	-NHCOCH_3_	H	5451.10	701.20	168.10	97.00
**7e**	-NHCOCH(CH_3_)_2_	H	333.40	39.60	203.5	94.00
**7f**	-NHCO(4-CH_3_C_6_H_4_)	H	43.00	39.00	30.00	106.00
**8a**	H	-NHCOCH_3_	8177.40	5401.30	209.50	17.00
**8b**	H	-NHCOCH(CH_3_)_2_	>100 000	>100000	207.50	141.60
**8c**	H	-NHCO(4-CH_3_C_6_H_4_)	9171.50	295.10	264.00	145.20
**8d**	-NHCOCH_3_	H	>100 000	855.10	971.10	>100 000
**8e**	-NHCOCH(CH_3_)_2_	H	>100 000	429.60	898.40	101.50
**8f**	-NHCO(4-CH_3_C_6_H_4_)	H	422.20	>100000	176.00	11.00
**9a**	H	-NHCOCH_3_	9036.70	>100000	2127.50	>100 000
**9b**	H	-NHCOCH(CH_3_)_2_	8661.20	57.10	300.0	>100 000
**9c**	H	-NHCO(4-CH_3_C_6_H_4_)	>100000	546.20	251.50	>100 000
**9d**	-NHCOCH_3_	H	>100 000	>100 000	1632.30	>100 000
**9e**	-NHCOCH(CH_3_)_2_	H	8495.20	73.20	29.00	201.50
**9f**	-NHCO(4-CH_3_C_6_H_4_)	H	>100 000	>100 000	1581.50	155.40
**SLC-0111** ^19^	–	–	5080.00	960.00	45.00	4.50
**AAZ**	–	–	250.00	12.10	25.70	5.70

The reported *K*_i_s values of SLC-0111.

*Mean from 3 different assays, by a stopped flow technique (errors were in the range of ± 5–10% of the reported values);

The following structure–activity relationship (SAR) was attained from the inhibition data listed in Table 1:

- The highly prevalent isoform hCA I was the highly inhibited isoform in this study with inhibition constants ranging from 33.00 nM to more than 100 000 nM. Compounds **7a**, **7b**, **7c**, and **7f** (*K*_i_s = 58.20, 56.30, 33.00 and 43.00 nM) showed significant activity ranging from 4 to 8 times more than AAZ (*K*_i_ = 250.00 nM). Moreover, compounds **7d** and **7e** have moderate inhibition activity towards hCAI isoform (*K*_i_s = 5451.10 and 333.40 nM), respectively.
  Obviously, replacement of the sulfamoyl moiety with its isosteres ethyl ester and carboxylic group reduced activity as demonstrated in compounds **8a–f** and **9a–f**. Compound **8a** and **8c** displayed mild inhibition activity with (*K*_i_s = 8177.40 and 9171.50 nM), compound **8f** has reasonable inhibitory activity with (*K*_i_ = 422.20 nM). While compounds **9a**, **9b**, and **9e** showed moderate inhibiting activity (*K*_i_s = 9036.70, 8661.20, and 8495.20 nM), others have inhibition range up to 100 µM. Noteworthy, the two most active compounds **7c** and **7f** against hCAI isoform were sulphonamide derivatives as ZBG with tolyl substitution at position 3 and 4, respectively, whereas position 3 was the most active one **7c** (*K*_i_=33.00 nM) while **7f** has *K*_i_ = 43.00 nM. Furthermore, other active compounds **7a** and **7b** (*K*_i_s = 58.20 and 56.30 nM), were sulphonamide derivatives with aliphatic amide substituent at position 3 of phenyl group as a tail, and replacement of substituent to position 4 as in compounds **7d** and **7e** moderately affected the inhibitory activity with *K*_i_s = 5451.10 and 333.40 nM.
- hCA II was affected efficiently by benzenesulfonamide derivatives as demonstrated by compounds **7a**, **7b**, that show selective and potent activity inhibition constants (*K*_i_s = 2.50, 2.10 nM), respectively, which is fivefold more active than AAZ. Whereas compounds **7c**, **7e**, and **7f** elicited significant activity (*K*_i_s = 56.60, 39.60, and 39.00 nM), respectively, relative to AAZ (*K*_i_ = 12.10 nM)
  Also, replacing the (-SO_2_NH_2_) moiety with its isostere (COOH) decreased the inhibitory action (**8a**, **8c**, **8d**, and **8e**) (*K*_i_s = 5401.30, 295.10, 855.10, and 429.60 nM), respectively. Grafting with various substituents at the position 3 of the benzamide has a significant influence on the inhibitory efficacy with the following order -NHCOCH(CH_3_)_2_< -NHCOCH_3_< -NHCO(4-CH_3_C_6_H_4_). Whereas changing the substituent to position 4 diminished the inhibitory effect of compounds **7d** and **7e** (*K*_i_s = 701.20, 39.60 nM) except compound **7f** where the activity (*K*_i_ = 39.00 nM).
  Interestingly, compounds **9b** and **9e** (-COOC_2_H_5_) showed significant activity (*K*_i_s = 57.10 and 73.20 nM), respectively. Replacement of sulphonamide moiety to ester analogue (-COOC_2_H_5_) **9a**, **9c**, **9d**, **9f** reduced the inhibitory effect to less than 100 nM while, compounds **9b** and **9e** showed moderate activity with *K*_i_s = 57.10 and 73.20 nM, respectively. Unfortunately, replacement of SO_2_NH_2_ with its isostere (COOH) did not show any significant activity.
- Concerning hCA IX, the tumour-associated isoform, it was as well considerably inhibited by the compounds **7c** and **7f** bearing (-SO_2_NH_2_) as ZBG (*K*_i_s = 31.20 and 30.00 nM) relative to AAZ (*K*_i_ = 25.70 nM).
  Surprisingly, replacing the (-SO_2_NH_2_) moiety with (COOC_2_H_5_) produced a slight increase in the inhibitory action as in compound **9e** (*K*_i_ = 29.00 nM). Although, replacing the (-SO_2_NH_2_) moiety with its (COOH) isostere in compounds **8a–f** did not reveal any significant inhibitory activity range (K_i_s = 176.00–971.10 nM).
  Noticeably, the inhibitory profiles of carbonic anhydrase in Table 1 suggested that only thioureido benzenesulfonamide bearing (3-(4-methyl benzamide)) (**7c)** or 4(4-methyl benzamide (**7f**), ester-based compound containing (3-(4-isobutyramidobenzoyl)) (**9e**) show a significant activity for the tumour-related hCA IX isoform.
- Regarding hCA XII, Compounds bearing benzoic acid moiety along with 3- acetamidobenzoyl **8a** (*K*_i_ = 17.00 nM) or 4-(4-methylbenzamide) **8f** (*K*_i_ = 11.00 nM) were the most effective among all the tested compounds Table 1.
  Furthermore, compounds containing sulphonamide as ZBG **7a–f** showed considerable inhibitory values as compounds **7d**, **7e**, and **7f** (*K*_i_s = 97.00, 94.00, and 106.00 nM, respectively). The inhibitory impact was decreased to 100 µM by replacing the sulphonamide moiety with (-COOC_2_H_5_) **9a–f**, with the exception of **9e** and **9f**, which display moderate activity (*K*_i_s = 201.50 and 155.40 nM).
- Finally, using a bioisosteric replacement strategy to replace the SLC-0111 ureido linker with a flexible thioureido one, as well as elongation strategies, the inhibitory activity against the hCA IX isoform was successfully increased. such as compounds **7c**, **7f**, and **9e** (*K*_i_ = 31.20, 30.00, and 29.00 nM), respectively, vs (*K*_i_ = 45.0 for SLC-0111)^44^ with selectivity index for inhibition of hCA IX over hCA I (*S*_I_ = 293 for **8e**) vs (*S*_I_ = 113 for SLC-0111) Table 2. Unfortunately, the increased activity towards hCA II was coupled by decreased activity against the hCA XII isoform, resulting in a worse hCA II/XII selectivity index for target sulphonamide compounds. On the other hand, acid isostere compounds especially **8a** and **8f** exhibited potent activity against tumour associated isoform hCA XII with *K*_i_s =17.00 and 11.00 nM (Table 1) and with selectivity index for inhibition of hCA XII over hCA II (*S*_I_ = 318 and >9091, respectively) vs SLC-0111 (SI = 213; Table 3). To improve hCA II/XII selectivity for other compounds, more structural changes are required.

**Table 2.** Calculated selectivity indexes (S.I.s) for inhibition of hCA IX over hCA I and hCA II isoforms for compounds **7c**, **7f**, **9e**, **SLC-0111**, and **AAZ**.

Compound No.	I/IX	II/IX
7c	1.0	2.0
7f	1.4	1.3
9e	293	2.5
SLC-0111	113	21
AAZ	10	0.5

**Table 3.** Calculated selectivity indexes (S.I.s) for inhibition of hCA XII over hCA I and hCA II isoforms for compounds **8a**, **8f**, **SLC-0111**, and **AAZ**.

Compound No.	I/XII	II/XII
8a	481	318
8f	38	>9091
SLC-0111^44^	1129	213
AAZ	44	2

### Molecular modeling study

#### Molecular docking

**Figure 3.**
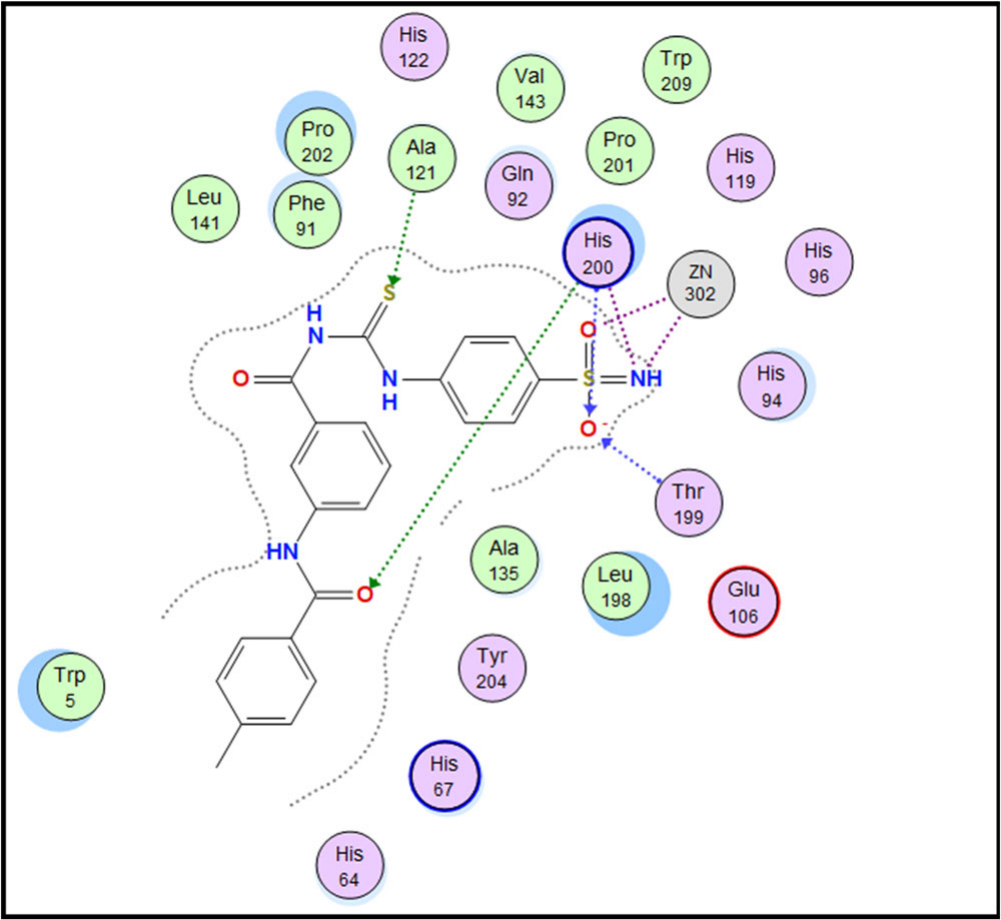
2D interaction of compound **7c** within the active site of hCAI.

**Figure 4.**
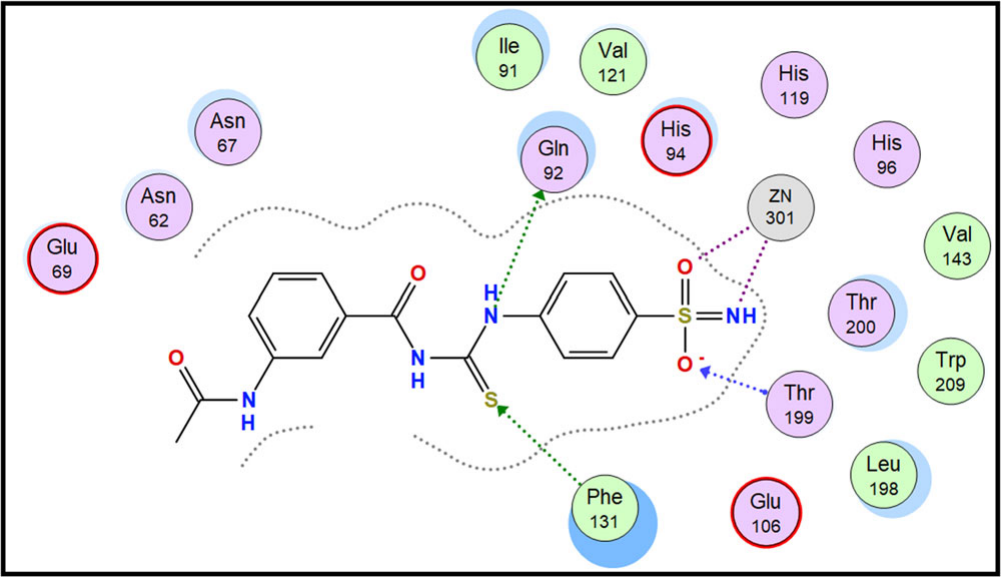
2D interaction of compound **7a** within the active site of hCAII.

**Figure 5.**
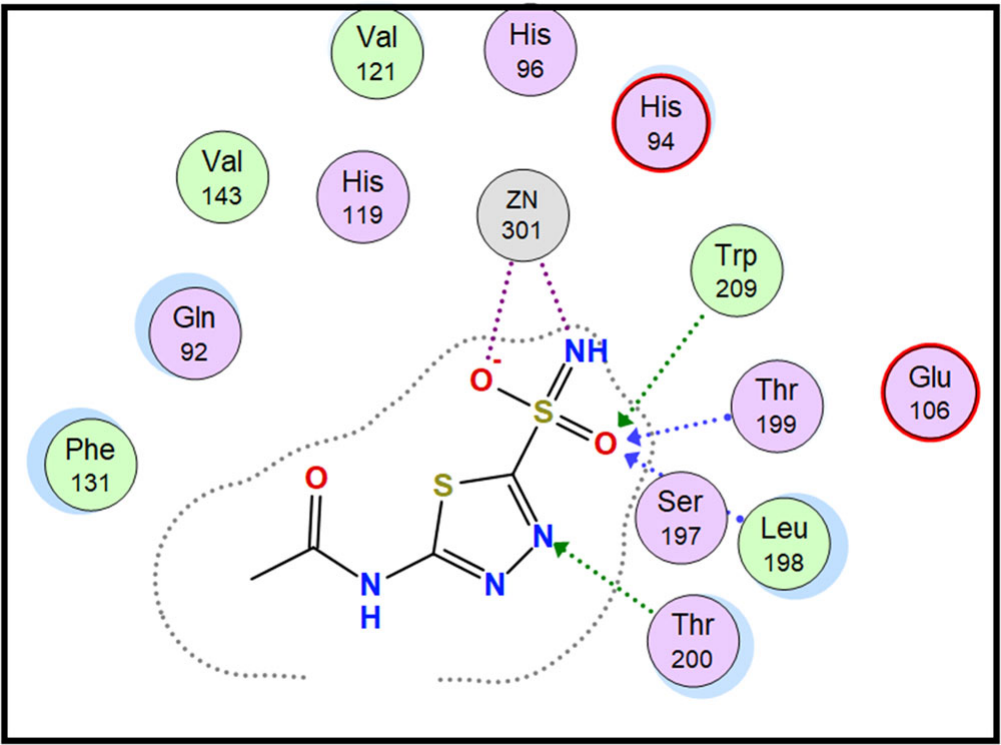
2D interaction of **AAZ** within the active site of hCAII.

**Figure 6.**
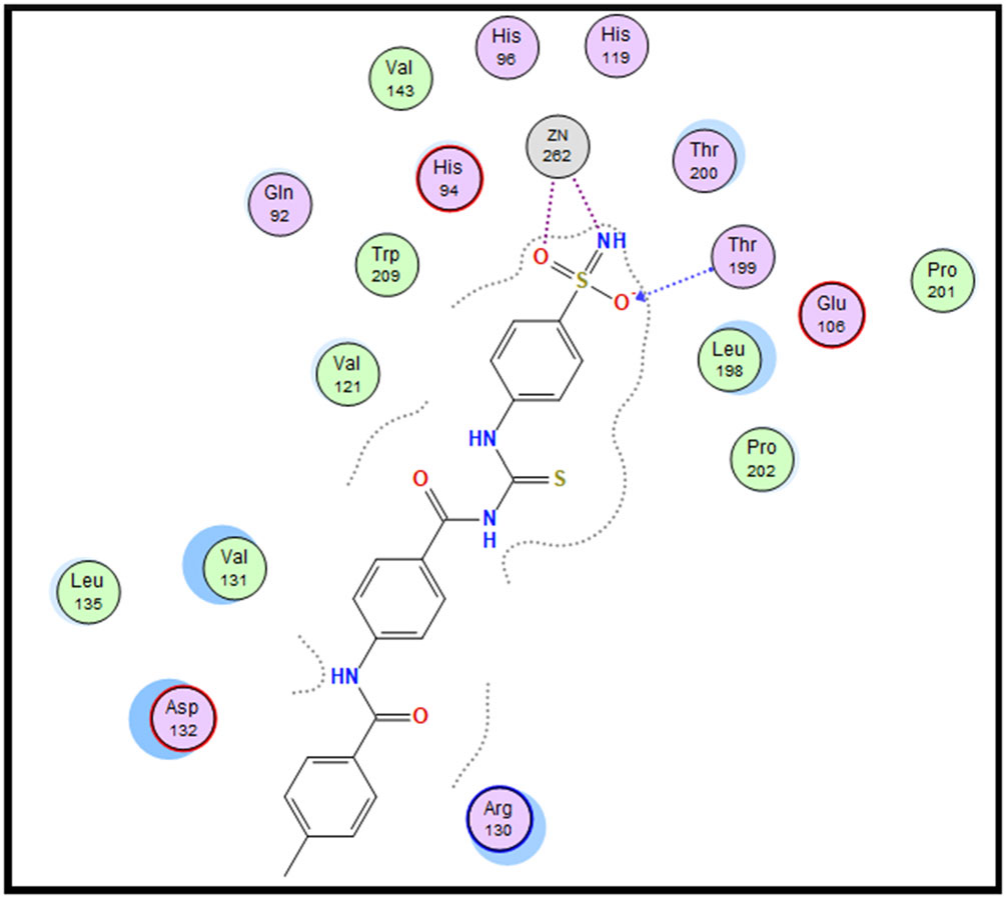
2D interaction of compound **7f** within the active site of hCAIX.

**Figure 7.**
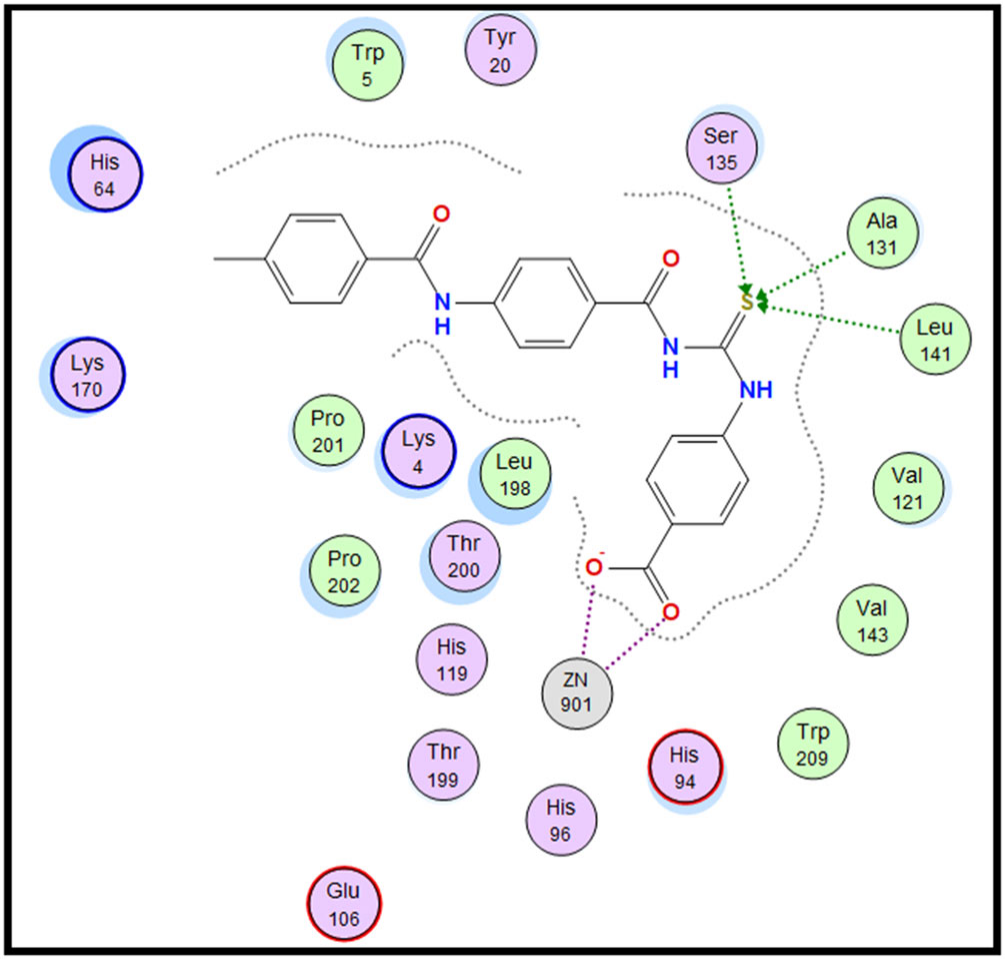
2D interaction of compound **8f** within the active site of hCAXII.

**Figure 8.**
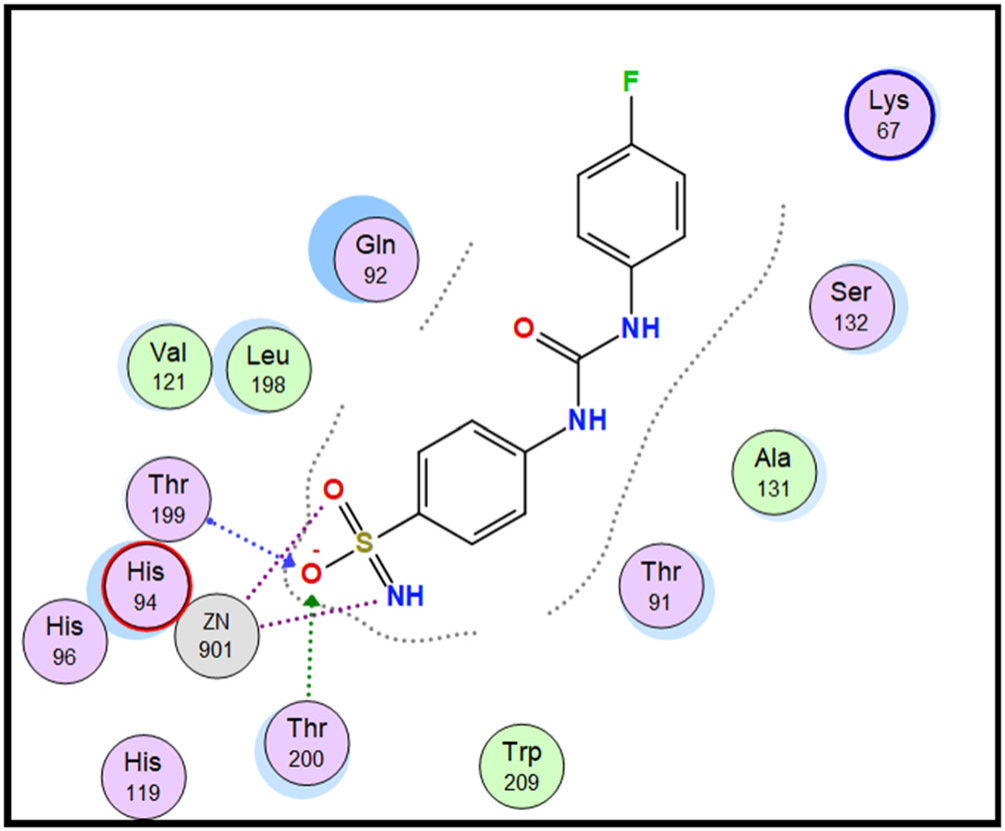
2D Interaction of **SLC-0111** within the active site of hCAXII.

Molecular docking is conducted for candidates **7c** and **7f** against hCAI, **7a** and **7b** against hCAII, **7f** and **9e** against hCAIX and **8a** and **8f** against hCAXII, to investigate their binding affinity in a correlation with their significant CA inhibitory activities. All of the targeted compounds coordinated with the Zn ion through the (O = S and NH) of sulphonamide (**7a**, **7b**, **7c**, and **7f**), (COO) of carboxylic acid (**8a** and **8f**) or (COOEt) (**9e**). As elaborated from the docking simulation, most of the benzenesulfonamide derivatives are engaged also in H-bonding with Thr199 through SO_2_ of sulphonamoyl except **7f** in hCAI, in addition to the hydrophobic interaction, and other residues that participate in binding such as His200, leu198, Ala131, Ser135 and leu141. Docking scores for the targeted compounds fall in the range of (−11.25 to −17.24 Kcal/mol) and were highly consistent with their inhibitory activity ranking against hCA isoforms (Figures 3–8).

2D diagram of other compounds are provided in the supplementary data.

#### Physicochemical, ADME, and pharmacokinetic properties prediction

The most active compounds **7a**, **7b**, **7c**, **7f**, **8a**, **8f**, and **9e** based on hCAs inhibitory activity results, are evaluated for their physicochemical characteristics as well as prognosis ADME parameters, pharmacokinetic properties, and drug-like nature using the Swiss ADME online web application, which is provided by the Swiss Institute of Bioinformatics (SIB). This is done to make sure that they are favourable congeners from both a pharmacokinetic perception as well as biological effect.

**Figure 9.**
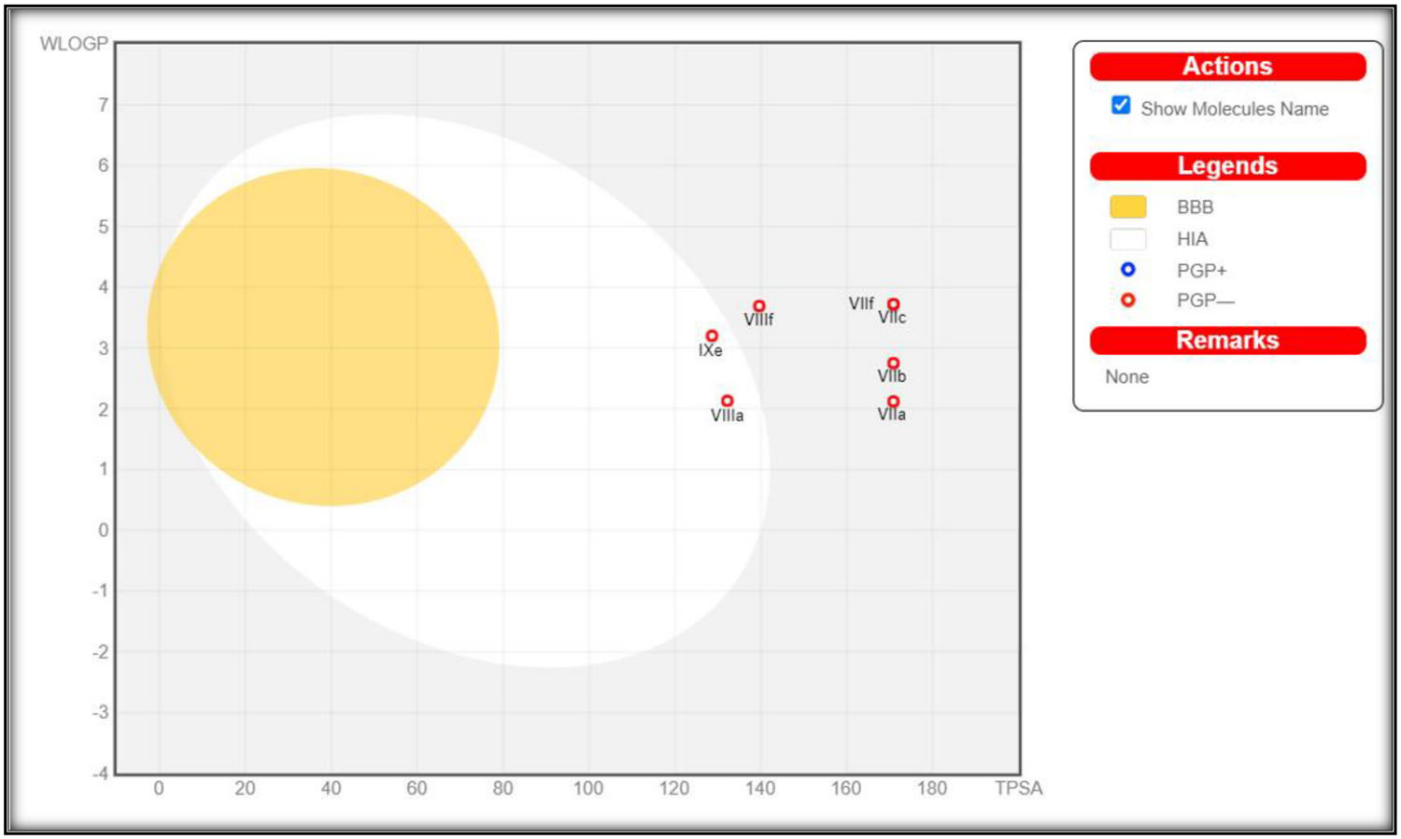
Expected boiled-egg plot from Swiss ADME online web tool for compounds **7a**, **7b**, **7c**, **7f**, **8a**, **8f**, and **9e**.

The presented compounds are expected to have promising physicochemical and pharmacokinetic characteristics. They exhibit a portended logPo/w in a range of 1.26–4.24, good water solubility for **7a**, **7b**, and **8a** compounds while **7c**, **7f**, **8f**, and **9e** compounds have moderate solubility, with high GIT absorption for **8a** and **9e**, while other compounds have low GIT absorption. No BBB permeability for all selected compounds, therefore no expected CNS side effects. Figure 9 demonstrates the BOILED-Egg plot of the WLOGP vs. TPSA (Topological Polar Surface Area)^45^^,^^46^ of the tested compounds.

Compounds **8a** and **9e** demonstrate a high human intestine absorption (HIA) area out of the seven compounds; they are expected to be absorbed in the gastrointestinal system due to their ideal physicochemical properties, which fall within the range of acceptable physicochemical parameters for oral bioavailability (GIT). While none of the selected compounds show blood-brain barrier permeability (BBB) which indicates their safety against CNS. The plot also demonstrates that none of the seven tested compounds are P-glycoprotein substrates (PGP-), and as a result, none of them are subject to the transporter’s efflux process, which many cancer cell lines use as a mechanism for drug resistance^47^^,^^48^. Furthermore, Swiss ADME revealed that all of the tested compounds, fulfil Lipinski’s (Pfizer)^49^ and Ghose’s (Amgen)^50^ the observed results predicted that the tested compounds have promising drug-likeness criteria. Nevertheless, they are not categorised as lead-like because their molecular weights exceed 350 and number of rotatable bonds is more than seven. Moreover, Swiss ADME data classified the seven most active compounds as non-PAINS (pan-assay interference compounds), notably the remarkable selectivity of our target compounds.

The computational analysis of the physicochemical and pharmacokinetic properties of the newly synthesised compounds revealed that the majority of them exhibit potential biological effectiveness and pharmacokinetic characteristics.

## Conclusion

The present study describes the design, synthesis, and carbonic anhydrase inhibitory activity of some benzoylthioureido benzenesulfonamide derivatives **7a–f** and their analogues **8a–f** and **9a–f** using replacement and elongation tail strategy of SLC-0111 aiming to increase potency and selectivity compared with acetazolamide AAZ as standard CAI. The synthesised compounds were assessed as CAIs against hCA I, II, IX, and XII. The benzenesulfonamides **7a**, **7b**, **7c**, **7e**, and **7f** bearing amide moiety at position 3 or 4 showed moderate to superior inhibitory activity against hCA I, II and IX with high selectivity towards hCA II isoforms, particularly the 3- acetamido derivative **7a** and 3-isobutyramido 7b (*K*_i_s = 2.50 and 2.10 nM), respectively, relative to that expressed by AAZ (*K*_i_s = 12.10 nM) and reported SLC-0111 (*K*_i_ =960.00 nM).

On the other hand, compounds **7a**, **7b**, **7c**, and **7f** exhibited a superior selectivity over hCAI (*K*_i_s =33.00–58.20 nM) compared with AAz (*K_i_* = 250.00 nM).

Despite of designing compounds **8a–f** as bioisosters to **7a–f**, only two compounds of the benzoic acid series **8a** and **8f** elicited a moderate inhibitory activity against hCAXII (*K*_i_s = 17.00 and 11.00 nM), respectively, in comparison to AAZ and reported SLC-0111 values (*K*_i_s = 5.70 and 4.50 nM), respectively. Compounds **7c**, **7f**, and **9e** were selective towards hCAIX (*K*_i_s =31.20, 30.00 and 29.00 nM) relative to AAZ and reported SLC-0111 (*K*_i_s =25.70 and 45.00, respectively). On the other hand, the CA inhibitory activity was reduced when SO_2_NH_2_ moiety was replaced by their analogues COOH or COOC_2_H_5_ with the exception of the ethylbenzoate derivative **9e** which displays a significant activity against hCAIX (*K*_i_ =29.00 nM). The best inhibitory activity was observed in compounds bearing the amide moieties at position 3.

In addition, Simulation of docking for the most active compounds **7a**, **7b**, **7c**, **7f**, **8a**, **8f**, and **9e** based on their docking binding patterns and scores, predicted their binding mode and binding affinity to the hCA I, II, IX, and XII active sites and explained their selectivity. Moreover, compounds **8a** and **9e** have favourable pharmacokinetic characteristics in addition to exhibiting significant CA inhibitory activity towards CA IX and XII respectively.

## Supplementary Material

Supplemental Material
