# Supplementary material for: Design and synthesis of some new benzoylthioureido benzenesulfonamide derivatives and their analogues as carbonic anhydrase inhibitors
Source: J Enzyme Inhib Med Chem. 2022 Oct 28;38(1):12–23. doi: 10.1080/14756366.2022.2132485 (PMC9621292; doi:10.1080/14756366.2022.2132485)
Supplement: Supplemental Material [file IENZ_A_2132485_SM9974.pdf]

Design and Synthesis of Some New Benzoylthioureido Benzenesulfonamide Derivatives and their Analogues as Carbonic Anhydrase Inhibitors

Khulood H. Oudah,[a] Walaa R. Mahmoud, [b] Fadi M. Awadallah,[b] Azza T.Taher,[c,d] Safinaz E-S Abbas,[b] Heba Abdelrasheed Allam,[b*] Daniela Vullo,[e*] Claudiu T. Supuran[e]

[a] *Pharmaceutical Chemistry department, College of Pharmacy, Al-Ayen University, Iraq*

[b] *Pharmaceutical Chemistry department, Faculty of Pharmacy, Cairo University, Kasr El-Eini Street, 11562, Cairo, Egypt.*

[c] *Department of Pharmaceutical Organic Chemistry, Faculty of Pharmacy, Cairo University, Kasr El-Eini Street, 11562, Cairo, Egypt.*

[d] *Department of Pharmaceutical Organic Chemistry, Faculty of Pharmacy, October 6 University(O6U), Giza, Egypt.*

[e] *Università degli Studi di Firenze, Department NEUROFARBA – Pharmaceutical and Nutraceutical section, University of Firenze, via Ugo Schiff 6, I-50019 Sesto Fiorentino, Firenze, Italy.*

Information Classification: General

^{1}H-NMR and ^{13}C-NMR spectra

Information Classification: General

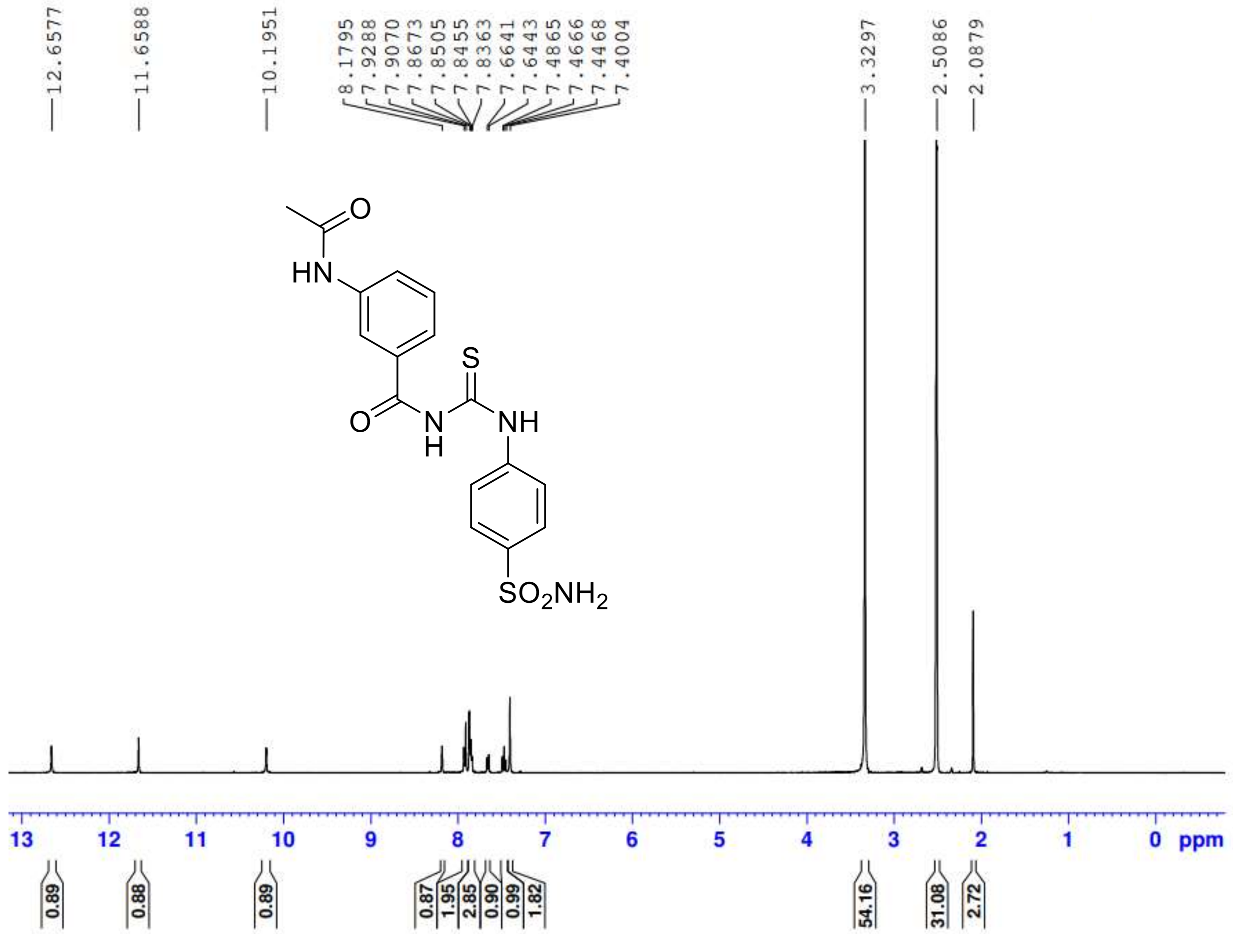

Figure 1: ^{1}H-NMR spectrum of 3-Acetamido-N-((4-sulfamoylphenyl)carbamothioyl)benzamide **7a**

Information Classification: General

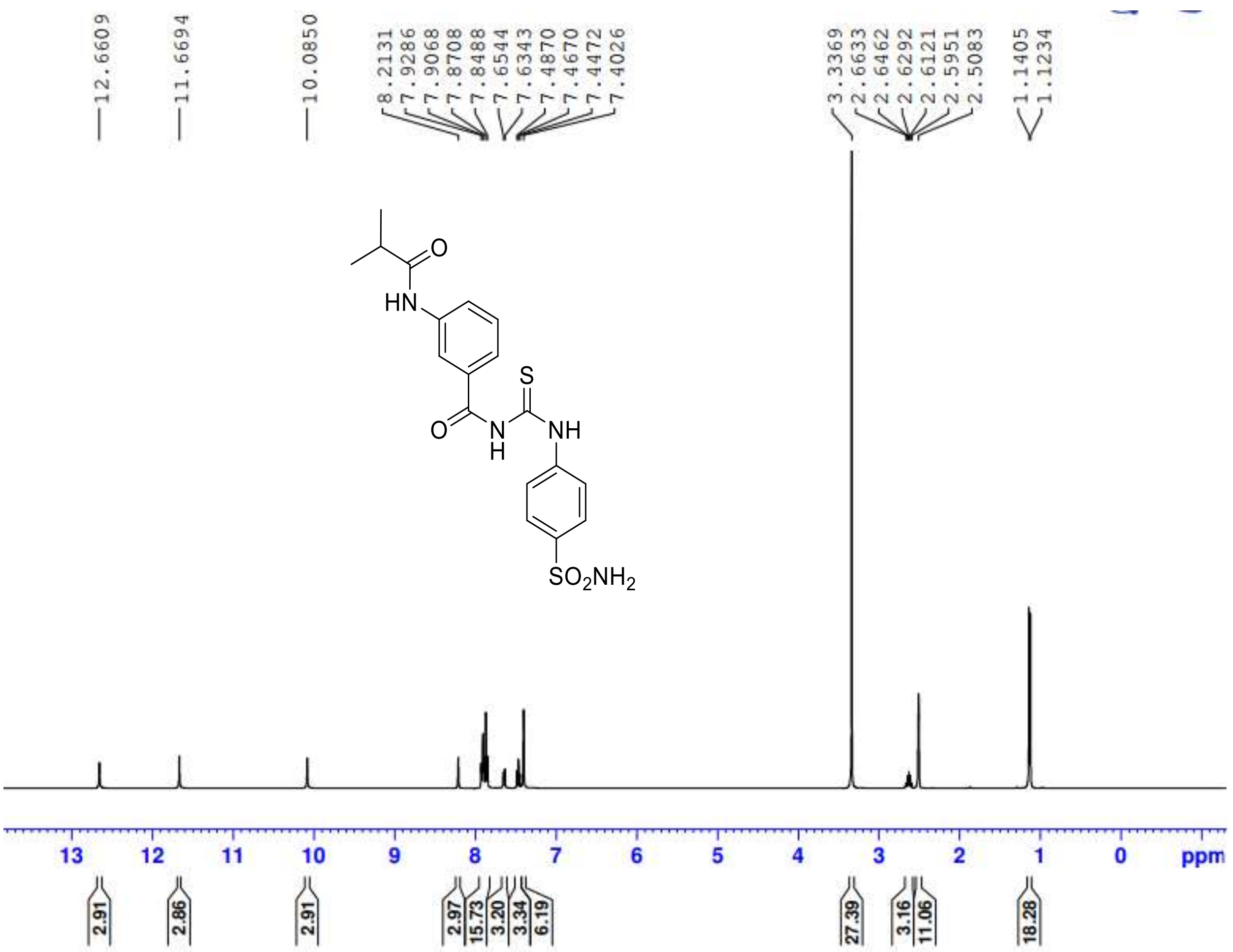

Figure 2: ^{1}H-NMR spectrum of 3-Isobutyramido-N-((4-sulfamoylphenyl)carbamothioyl) benzamide**7b**

Information Classification: General

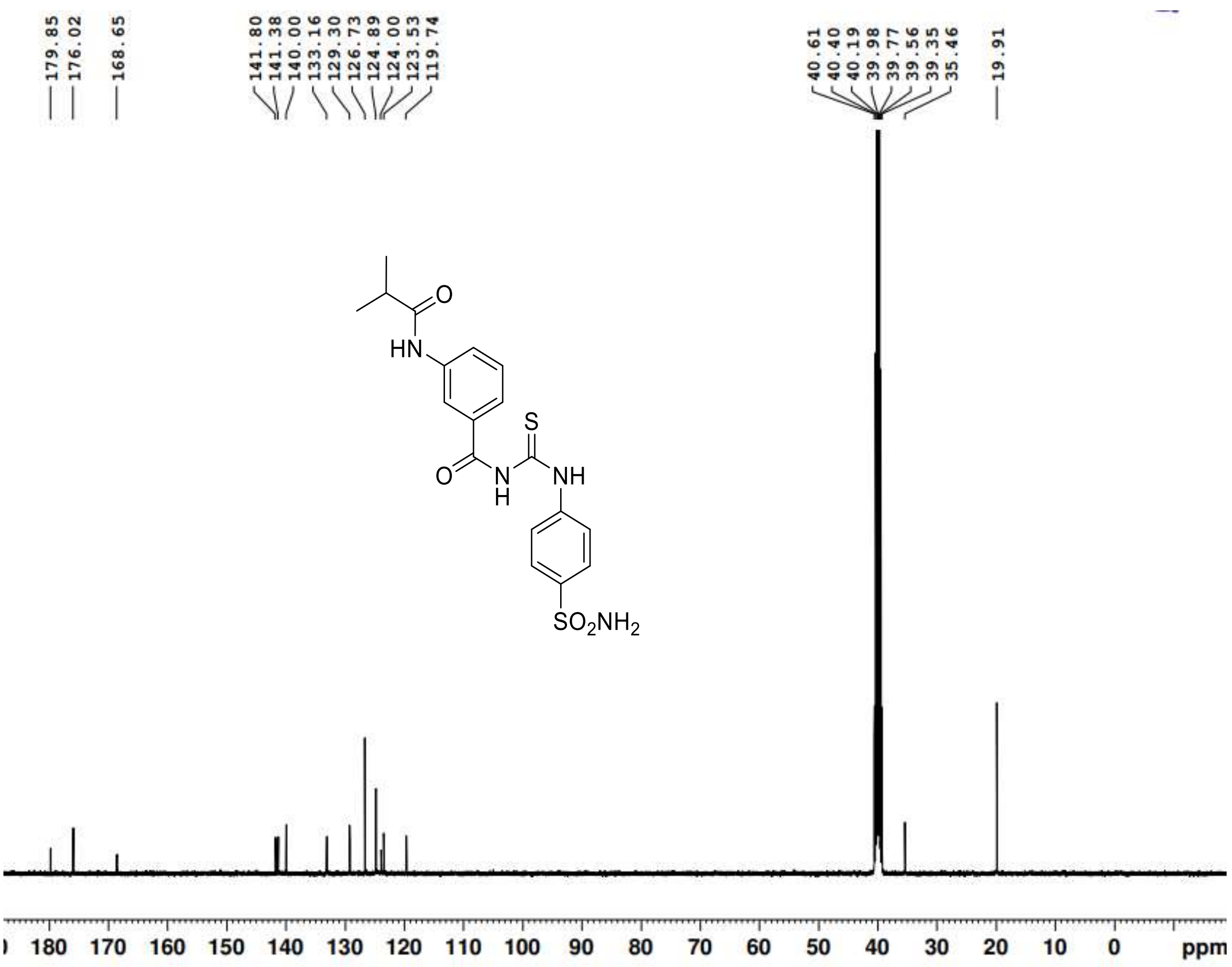

Figure 3: ^{13}C-NMR spectrum of 3-Isobutyramido-N-((4-sulfamoylphenyl)carbamothioyl) benzamide**7b**

Information Classification: General

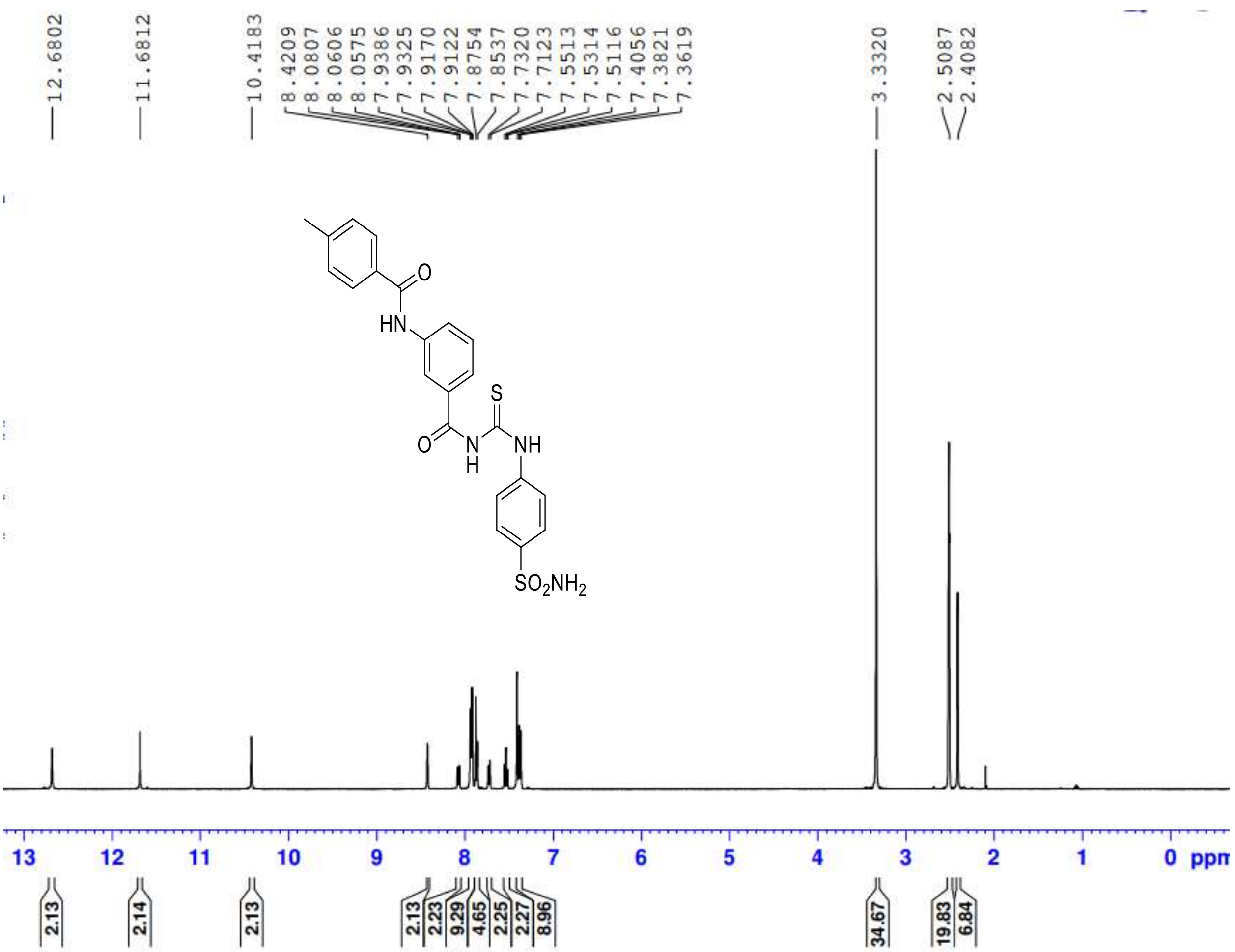

Figure 4: ^{1}H-NMR spectrum of 3-(4-Methylbenzamido)-N-((4-sulfamoylphenyl)carbamothioyl) benzamide **7c**

Information Classification: General

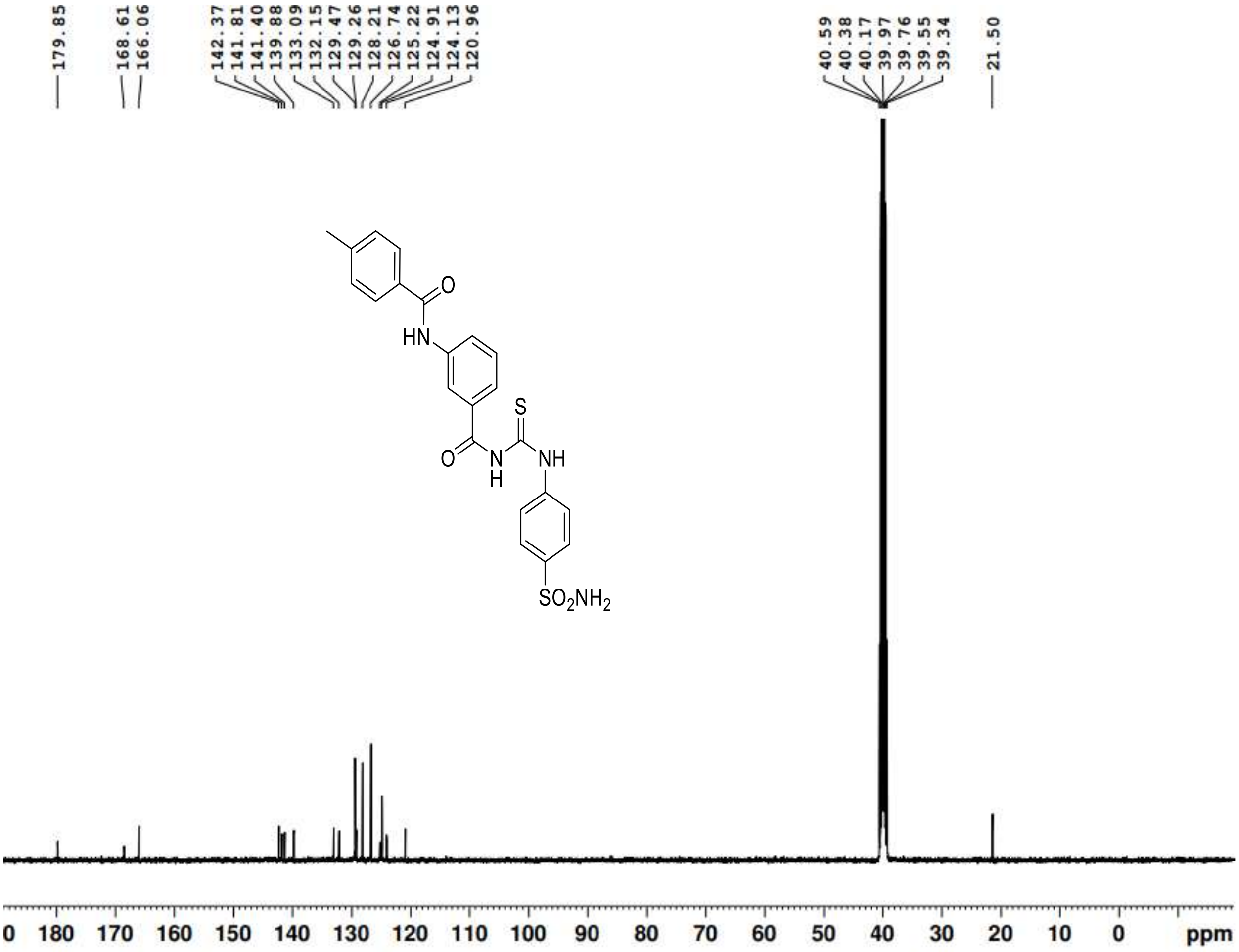

Figure 5: ^{13}C-NMR spectrum of 3-(4-Methylbenzamido)-N-((4-sulfamoylphenylcarbamothioyl) benzamide **7c**

Information Classification: General

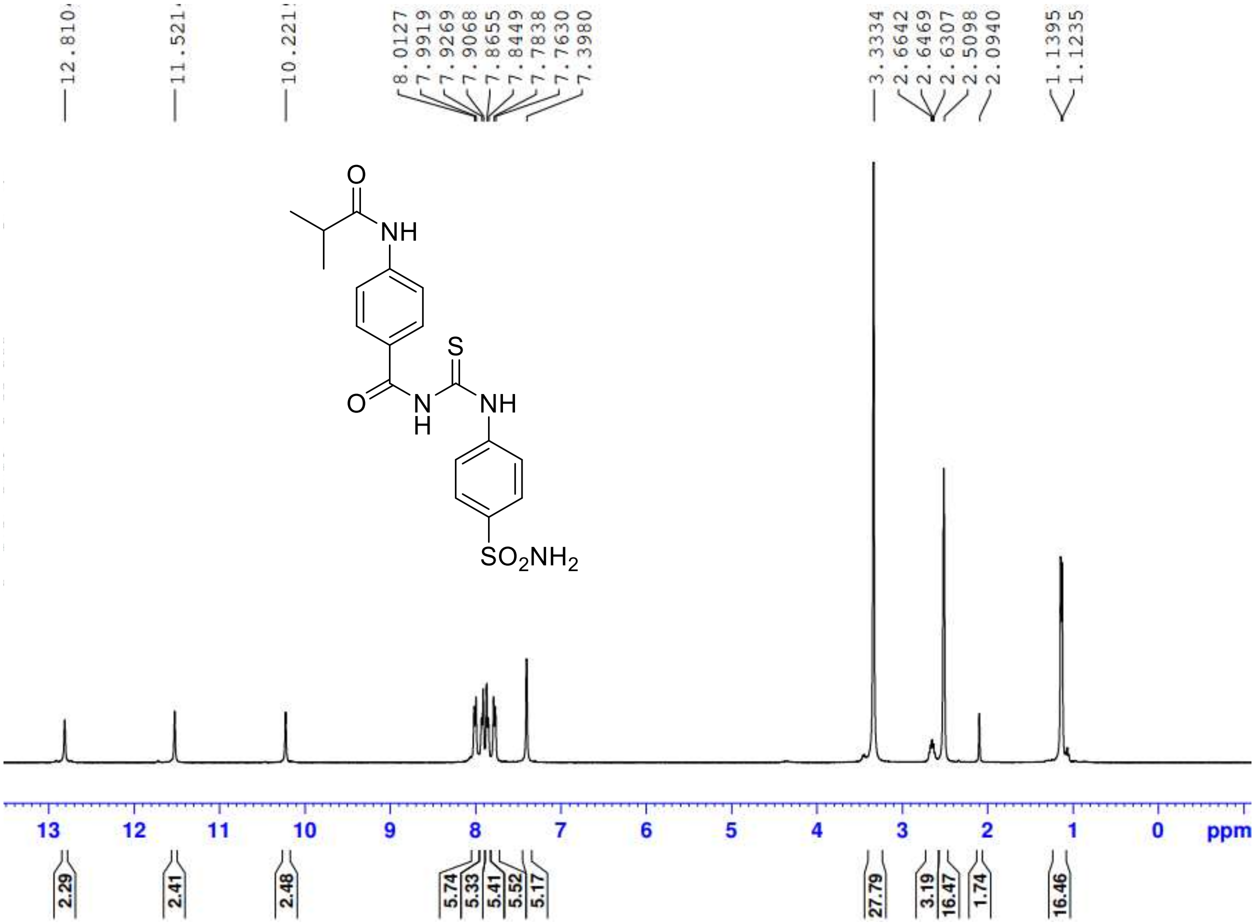

Figure 6: ^{1}H-NMR spectrum of 4-Isobutyramido-N-((4-sulfamoylphenyl)carbamothioyl) benzamide **7e**

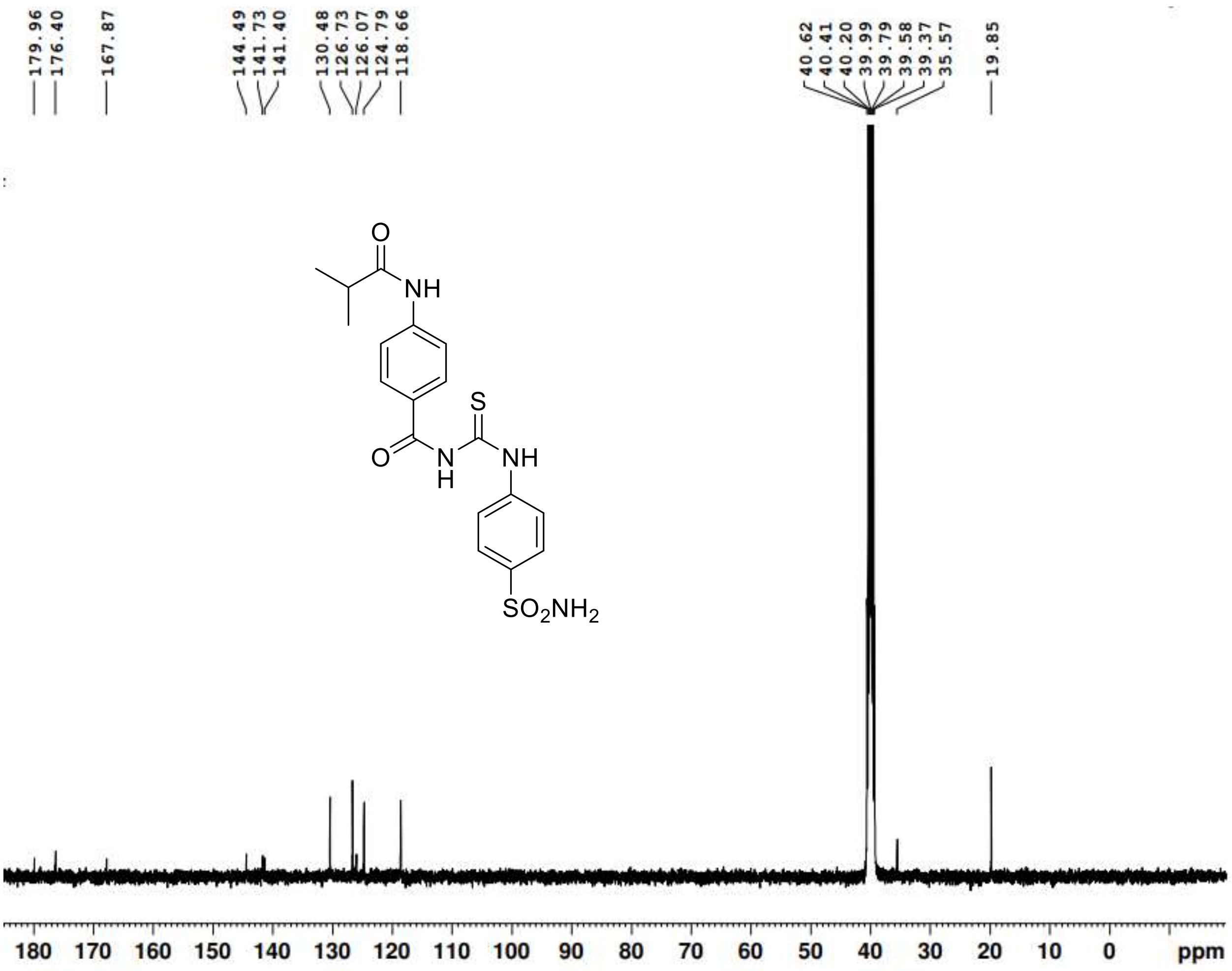

Figure 7: ^{13}C-NMR spectrum of 4-Isobutyramido-N-((4-sulfamoylphenyl)carbamothioyl) benzamide **7e**

Information Classification: General

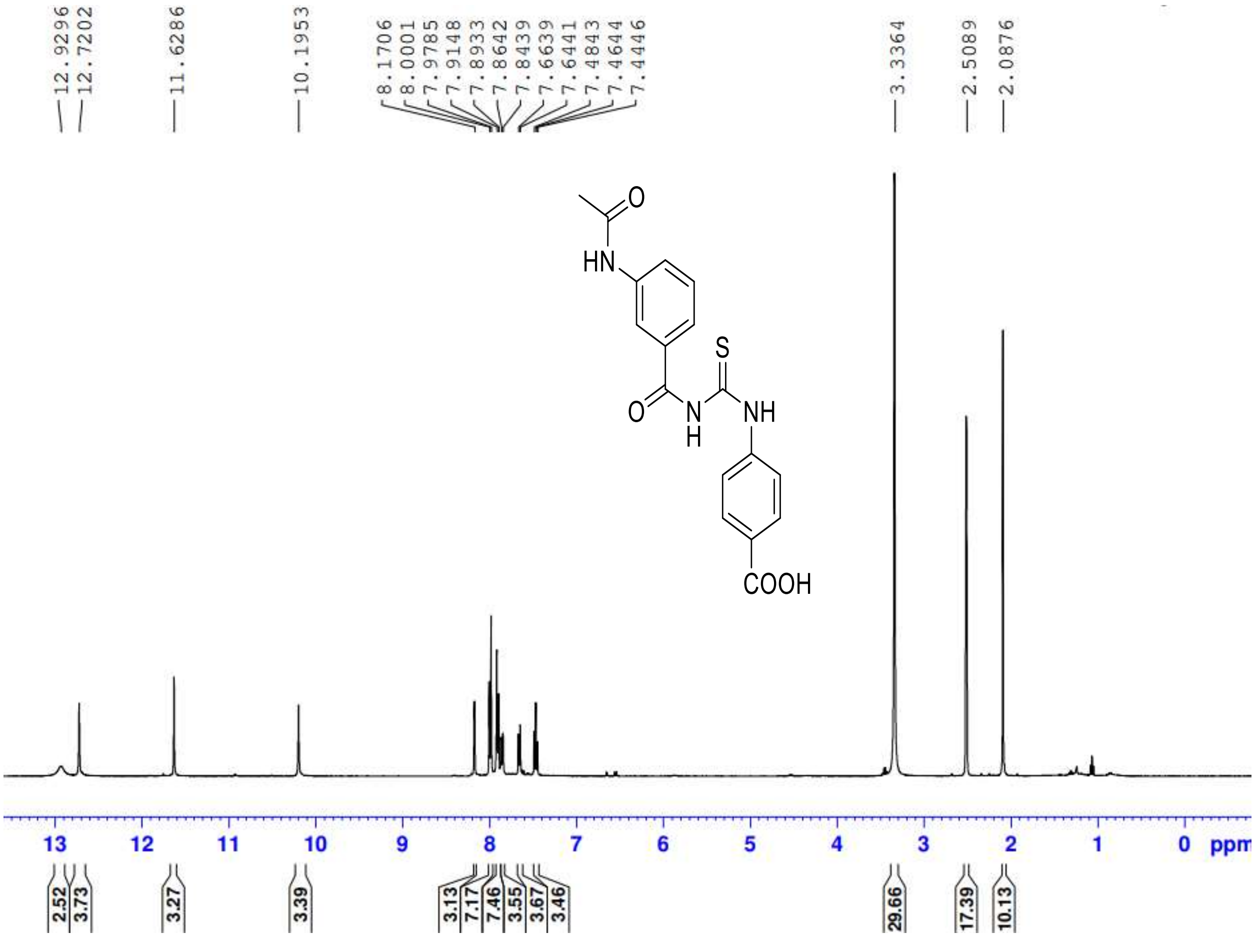

Figure 8: ^{1}H-NMR spectrum of 4-(3-(3-Acetamidobenzoyl)thioureido)benzoic acid **8a**

Information Classification: General

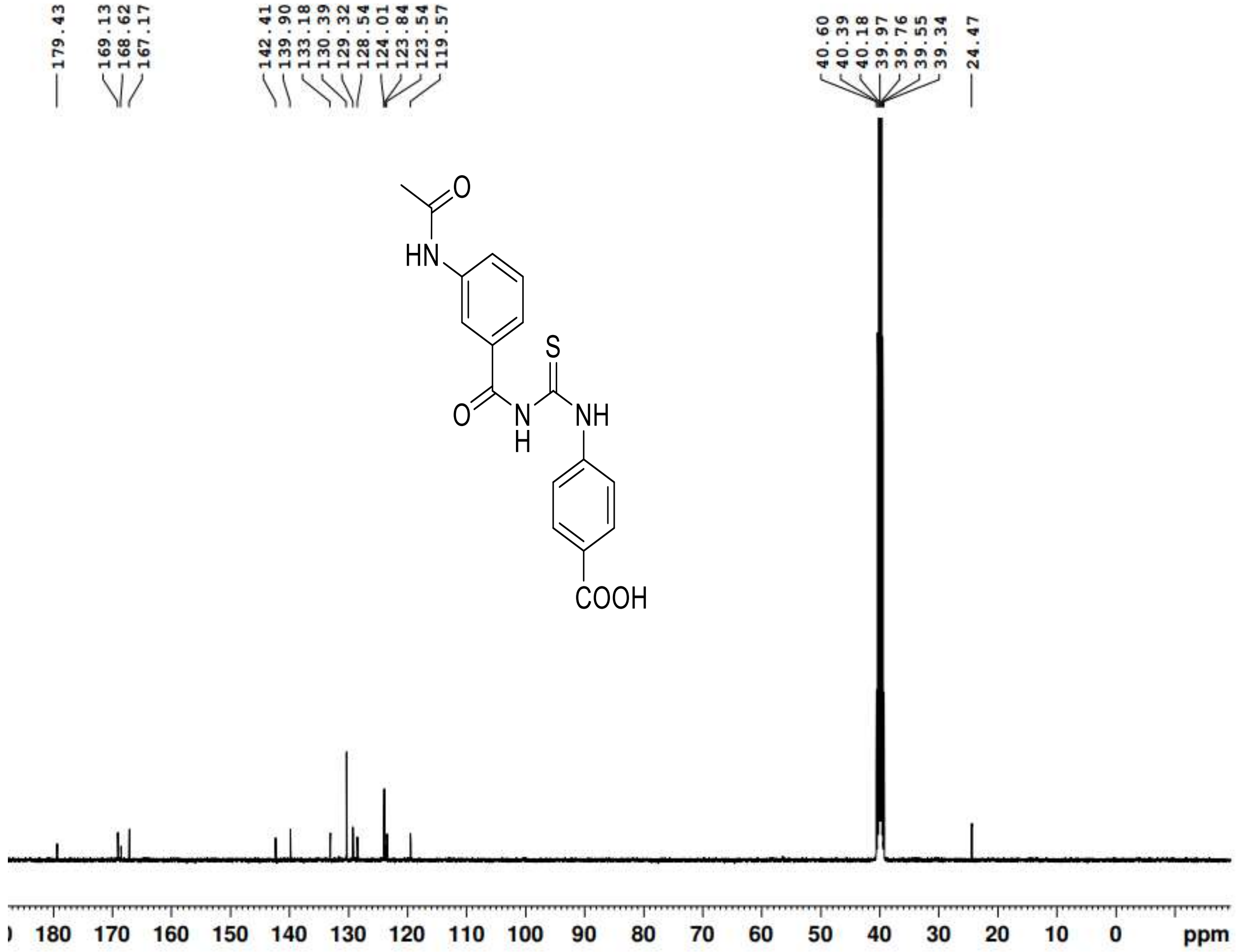

Figure 9: ^{13}C-NMR spectrum of 4-(3-(3-Acetamidobenzoyl)thioureido)benzoic acid **8a**

Information Classification: General

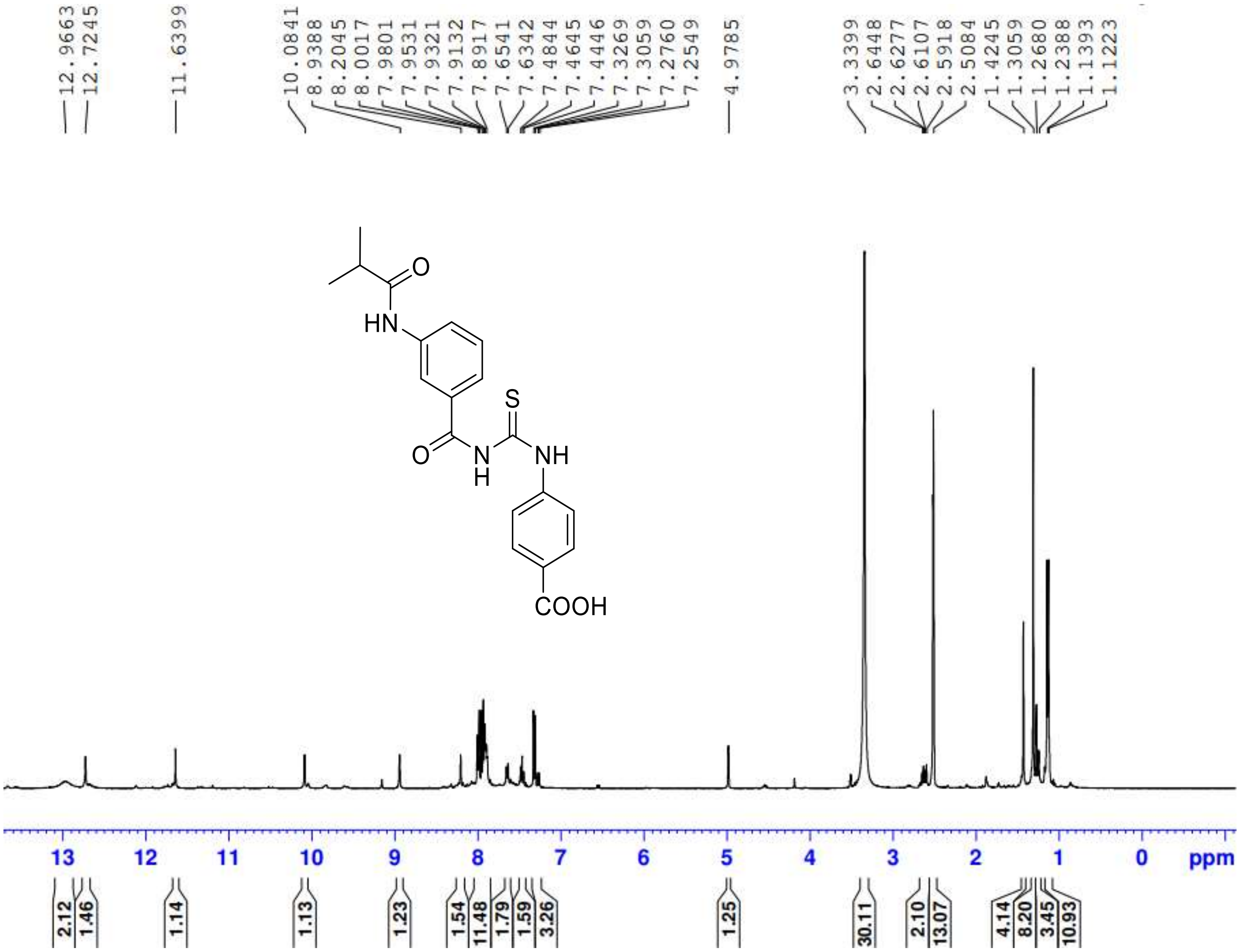

Figure 10: ^{1}H-NMR spectrum of 4-(3-(3-Isobutyramidobenzoyl)thioureido)benzoic acid **8b**

Information Classification: General

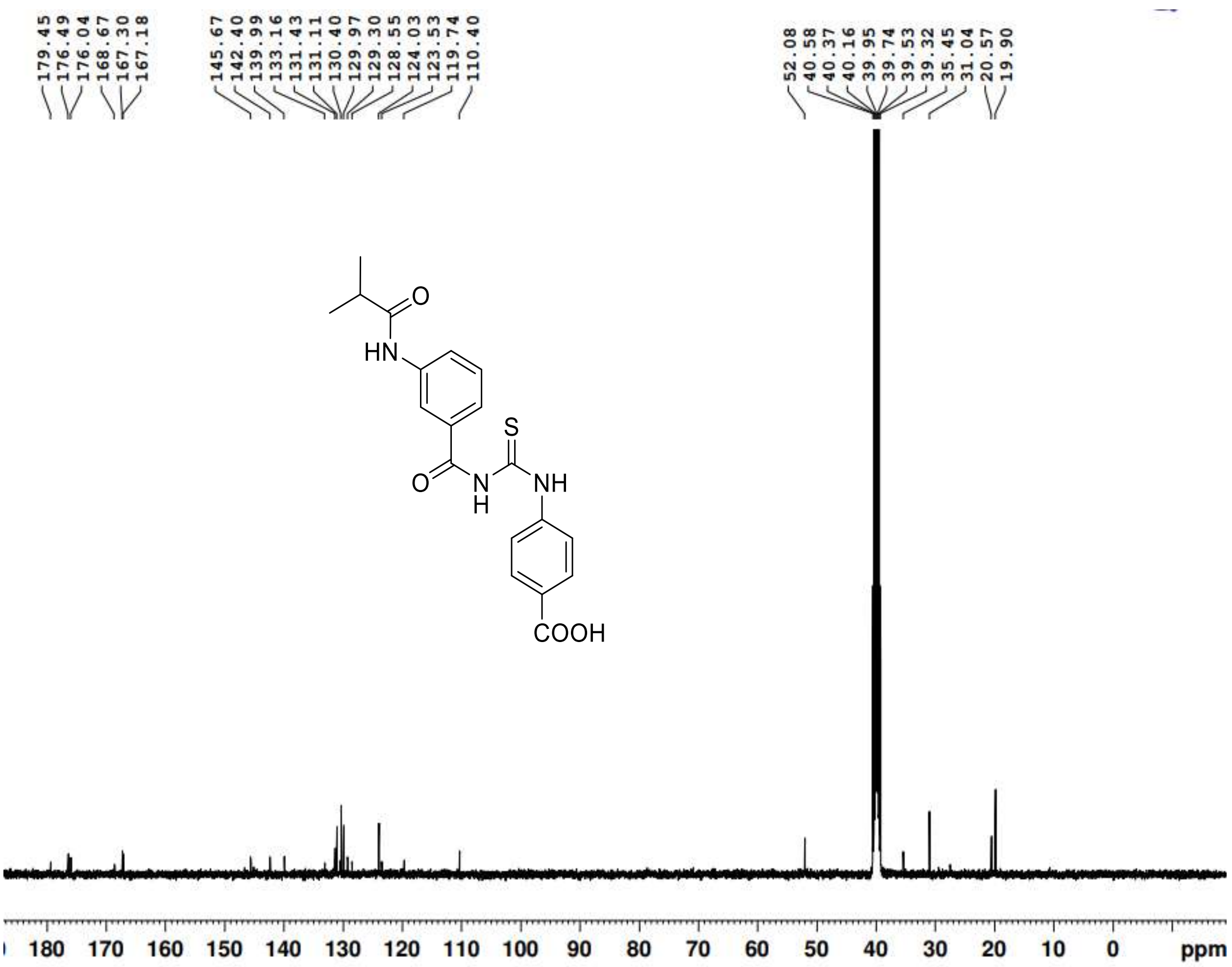

Figure 11: ^{13}C-NMR spectrum of 4-(3-(3-Isobutyramidobenzoyl)thioureido)benzoic acid **8b**

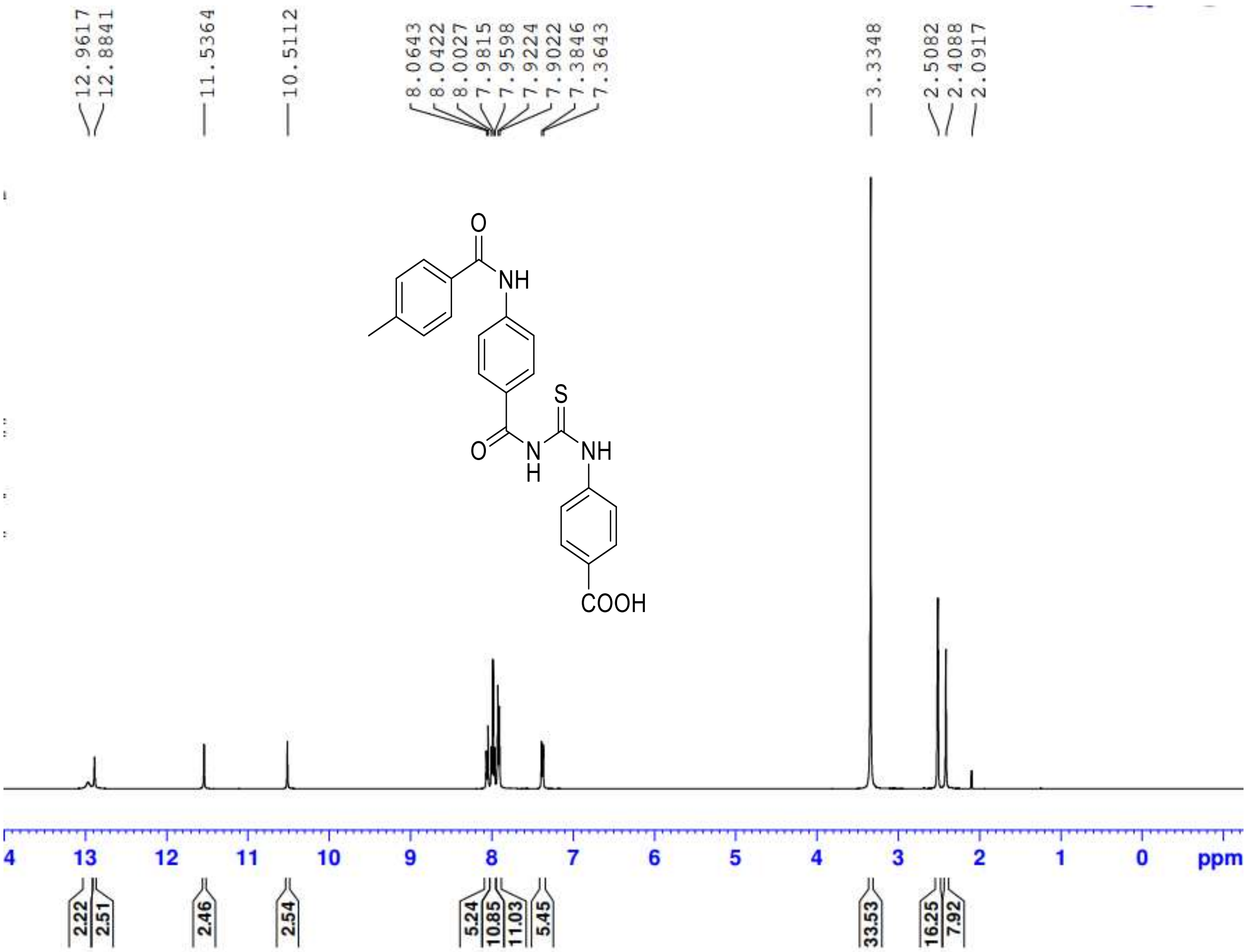

Figure 12: ^{1}H-NMR spectrum of 4-(3-(4-(4-Methylbenzamido)benzoyl)thioureido)benzoic acid **8f**

Information Classification: General

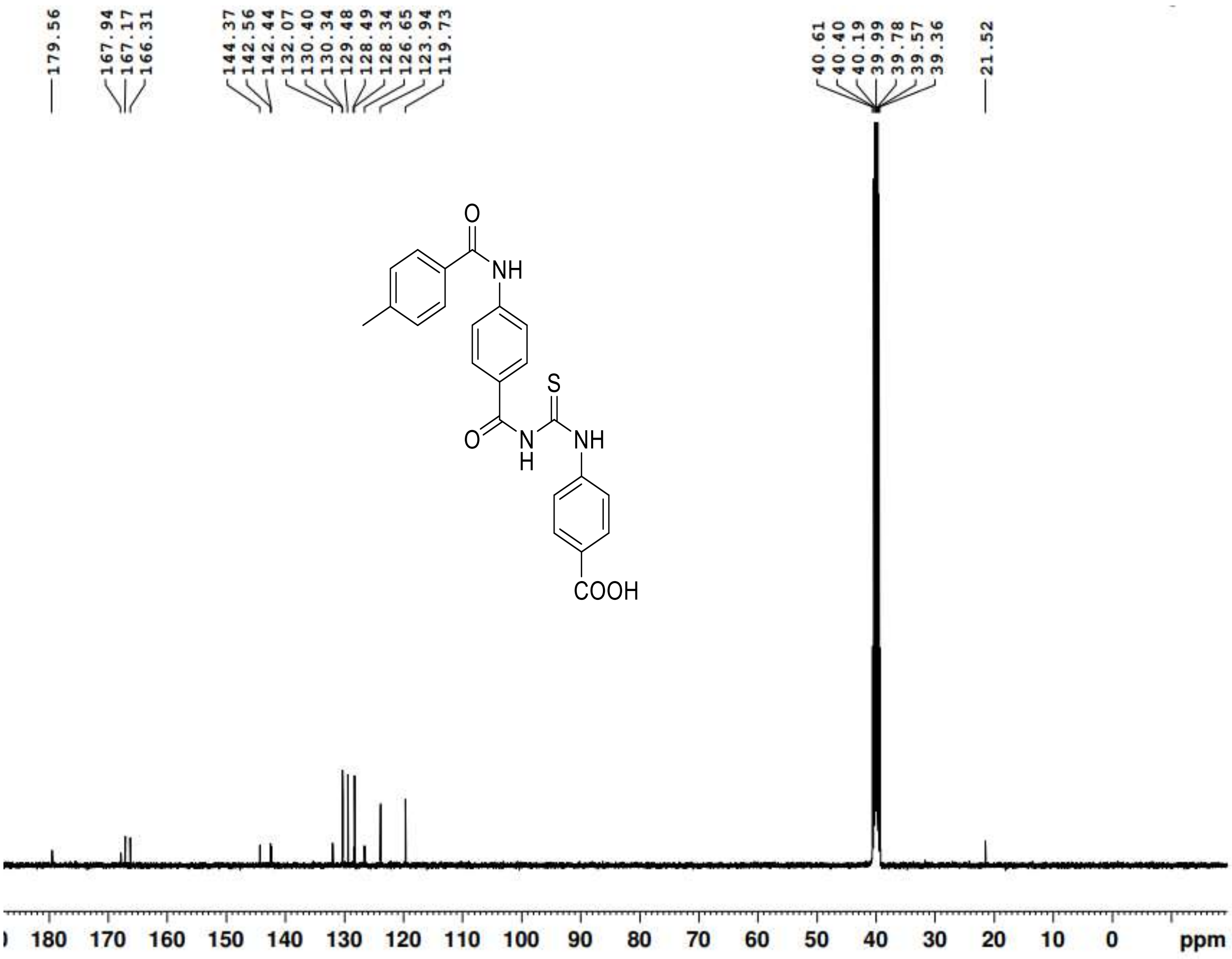

Figure 13: ^{13}C-NMR spectrum of 4-(3-(4-(4-Methylbenzamido)benzoyl)thioureido)benzoic acid **8f**

Information Classification: General

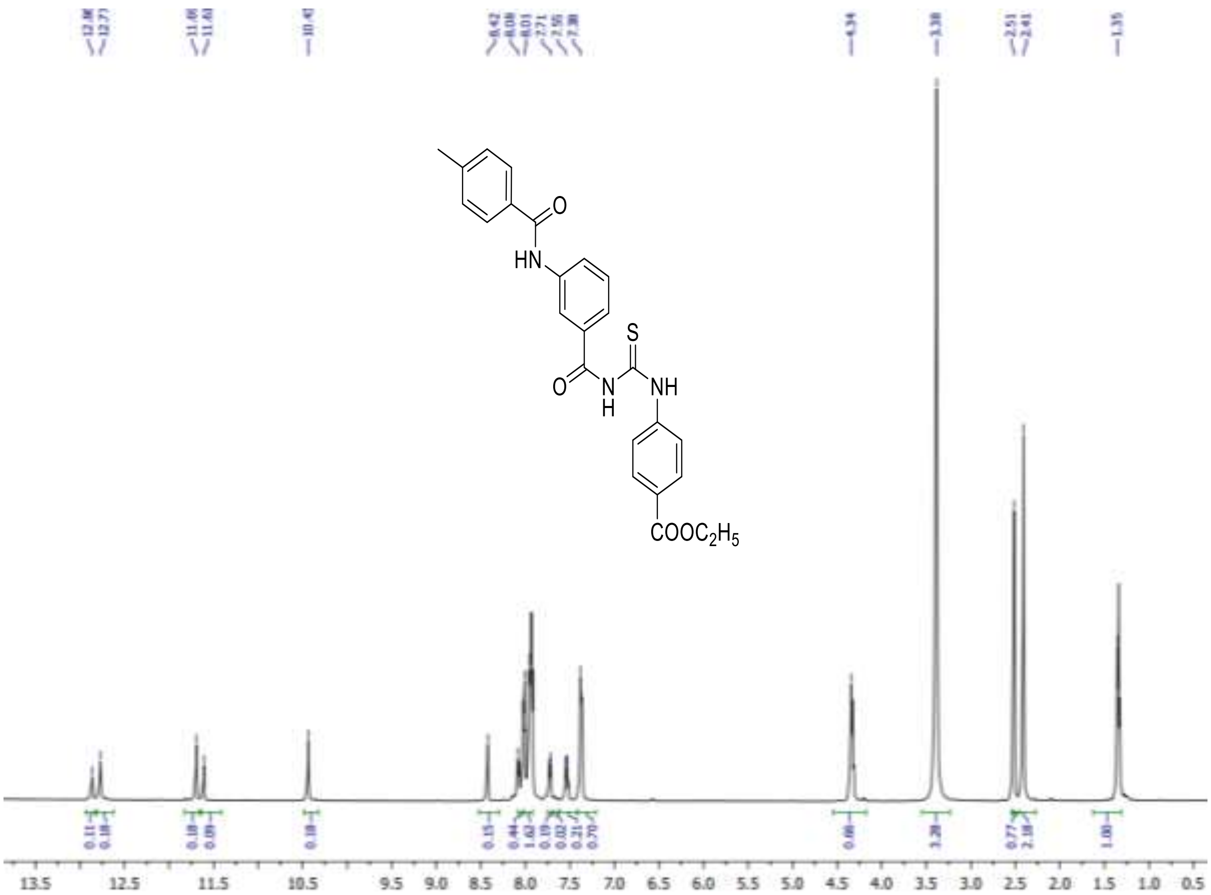

Figure 14: ^{1}H-NMR spectrum of Ethyl 4-(3-(3-(4-methylbenzamido)benzoyl)thioureido) benzoate **9c**

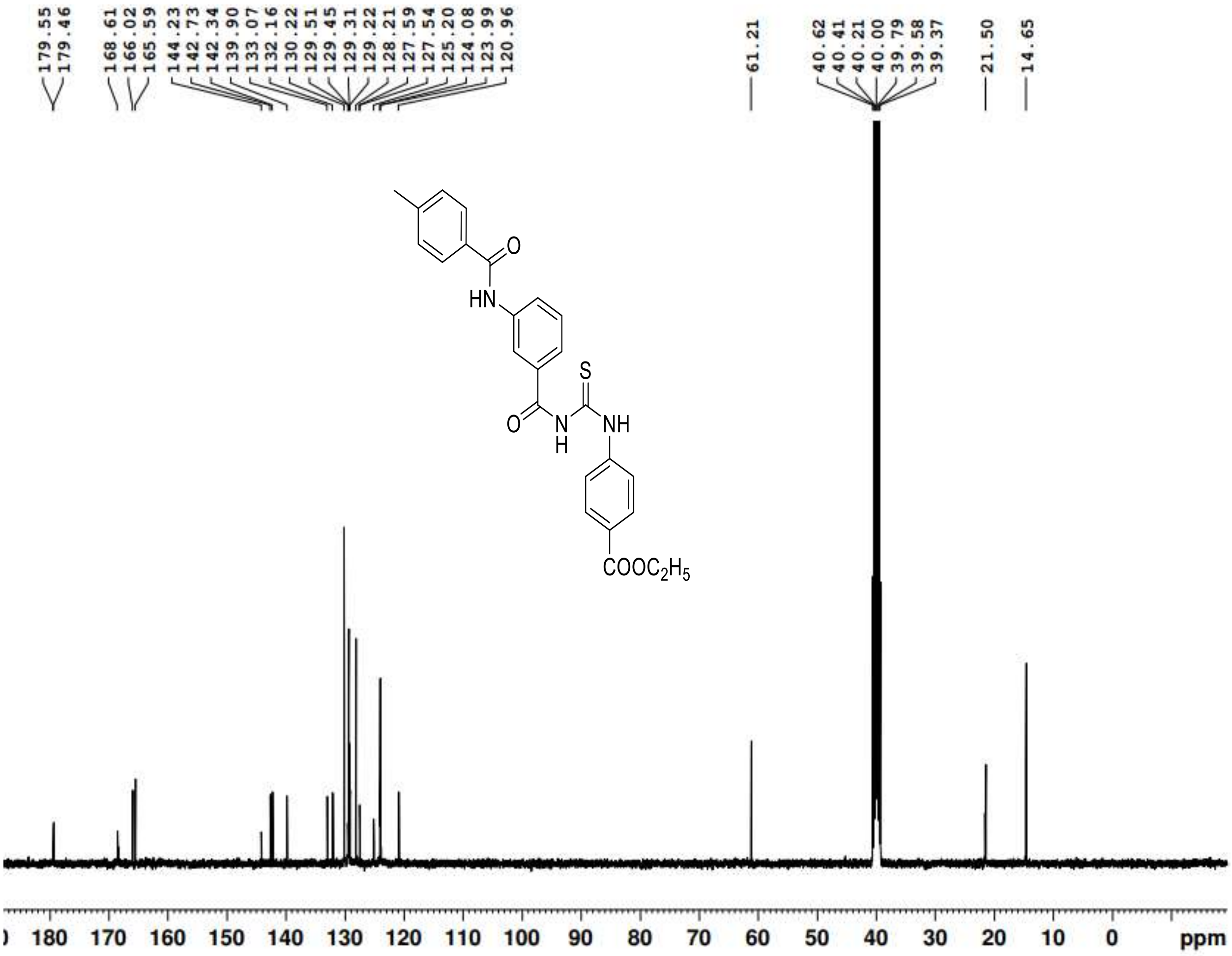

Figure 15: ^{13}C-NMR spectrum of Ethyl 4-(3-(3-(4-methylbenzamido)benzoyl)thioureido) benzoate **9c**

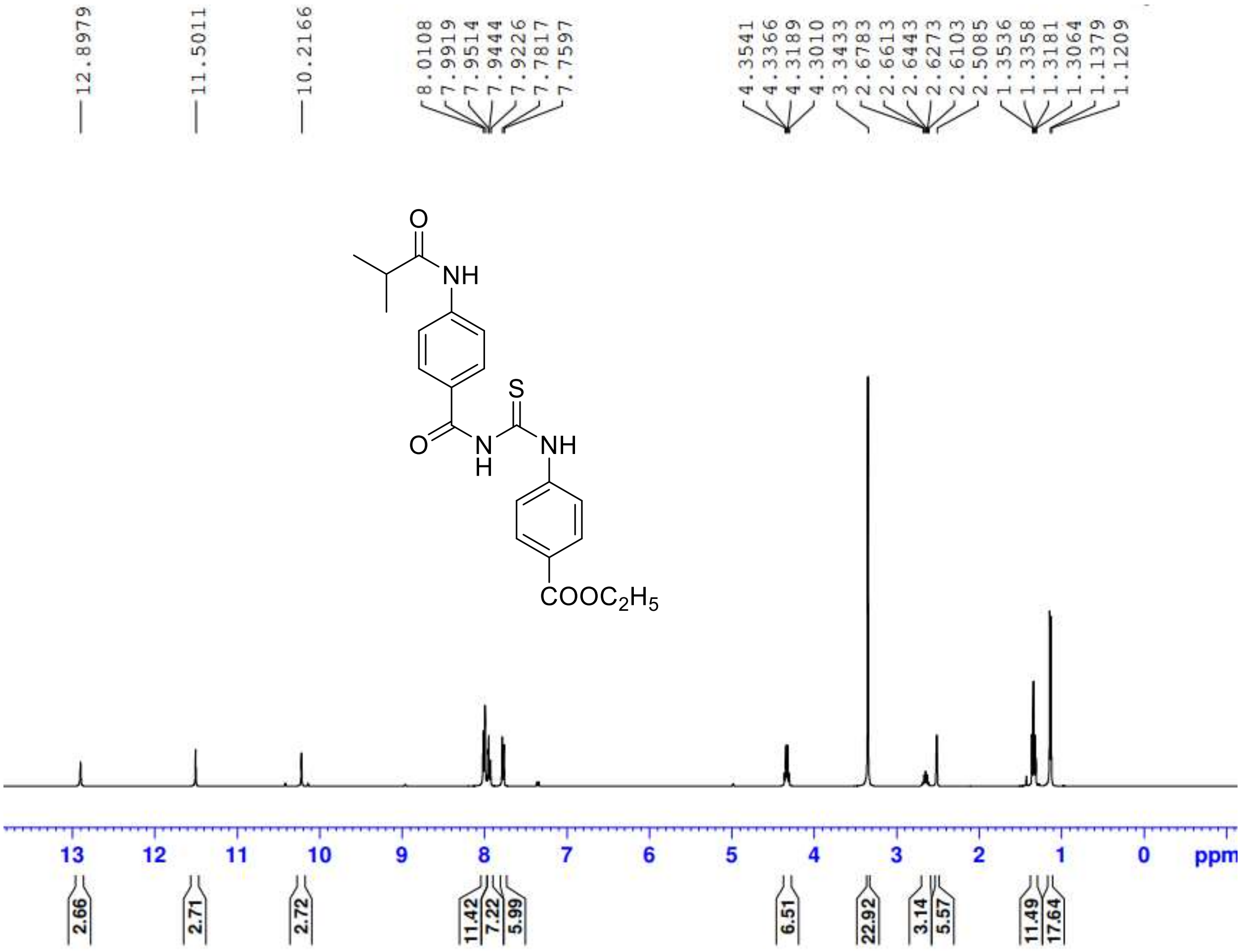

Figure 16: ^{1}H-NMR spectrum of Ethyl 4-(3-(4-isobutyramidobenzoyl)thioureido)benzoate **9e**

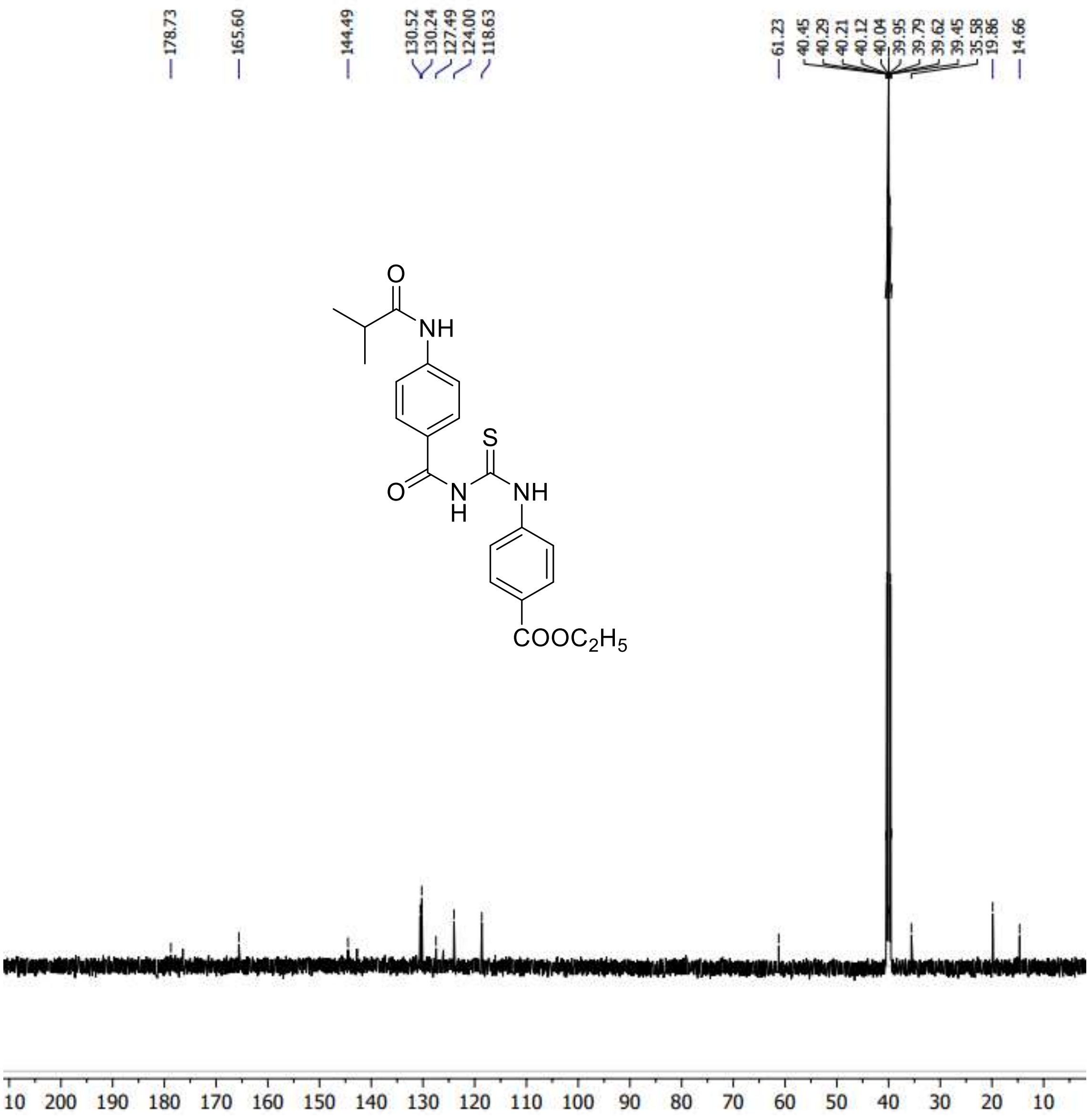

Figure 17: ^{13}C-NMR spectrum of Ethyl 4-(3-(4-isobutyramidobenzoyl)thioureido)benzoate **9e**

Information Classification: General

The 2D representations of the docking poses of the remaining promising compounds

Information Classification: General

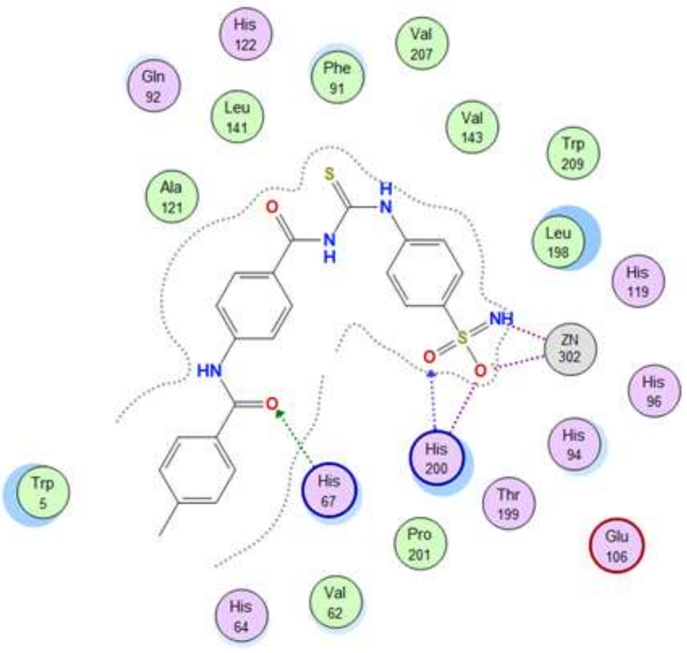

Figure18: 2D Interaction of compound 7f within the active site of hCAI

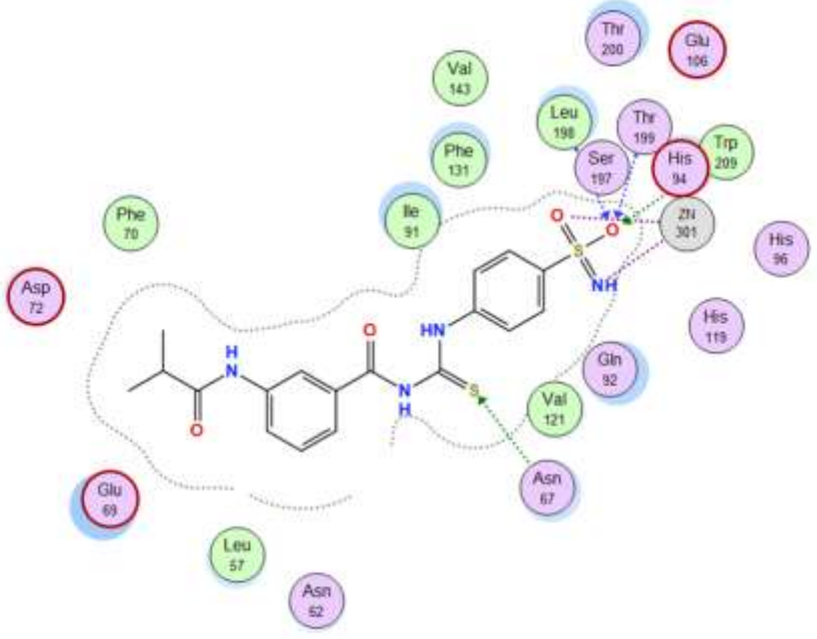

Figure19:2D Interaction of compound 7b within the active site of hCAII

Information Classification: General

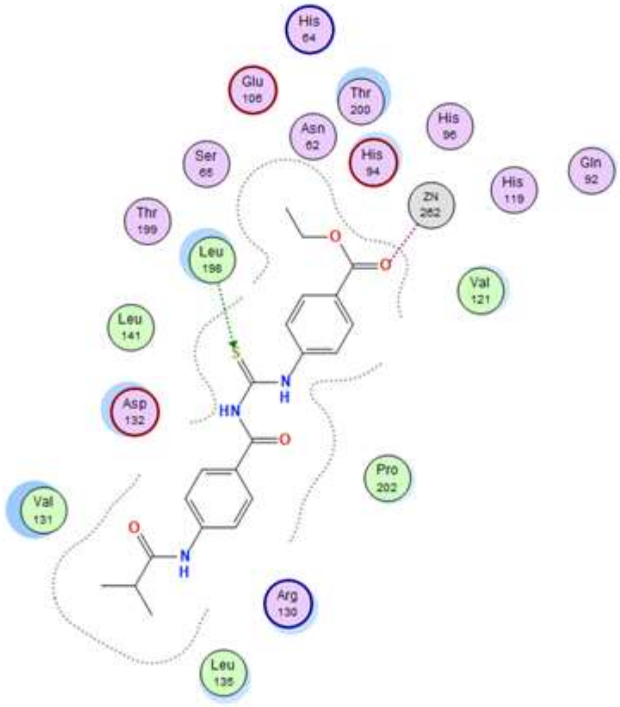

Figure20: 2D Interaction of compound 9e within the active site of hCAIX

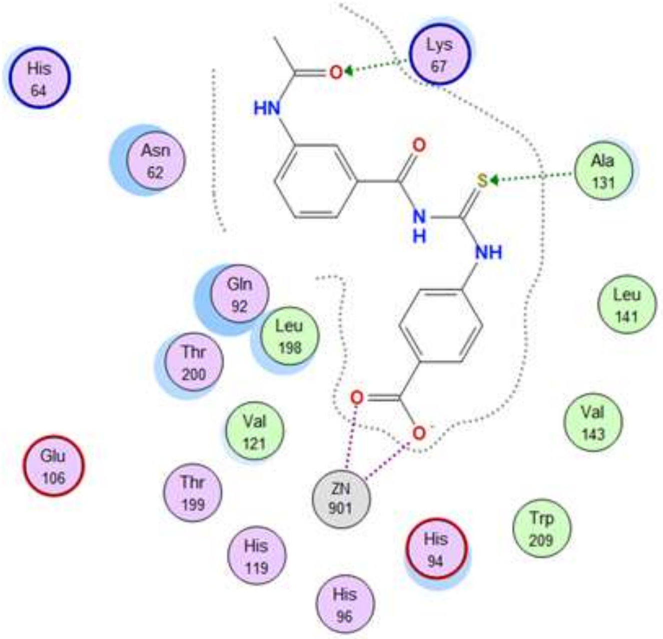

Figure21: 2D Interaction of compound 8a within the active site of hCAXII
